# Disturbed Vitamin A Metabolism in Non-Alcoholic Fatty Liver Disease (NAFLD)

**DOI:** 10.3390/nu10010029

**Published:** 2017-12-29

**Authors:** Ali Saeed, Robin P. F. Dullaart, Tim C. M. A. Schreuder, Hans Blokzijl, Klaas Nico Faber

**Affiliations:** 1 Department of Gastroenterology and Hepatology, University Medical Center Groningen, University of Groningen, 9713 GZ Groningen, The Netherlands; a.saeed@umcg.nl (A.S.); t.c.m.a.schreuder@umcg.nl (T.C.M.A.S.); h.blokzijl@umcg.nl (H.B.); 2 Institute of Molecular Biology & Bio-Technology, Bahauddin Zakariya University, Multan 60800, Pakistan; 3 Department of Endocrinology, University Medical Center Groningen, University of Groningen, 9713 GZ Groningen, The Netherlands; r.p.f.dullaart@umcg.nl; 4 Department of Laboratory Medicine, University Medical Center Groningen, University of Groningen, 9713 GZ Groningen, The Netherlands

**Keywords:** non-alcoholic fatty liver disease, metabolic syndrome, vitamin A, retinyl esters, retinol, retinoic acid, retinol binding protein 4, hepatic stellate cells, nuclear receptors, lipid metabolism

## Abstract

Vitamin A is required for important physiological processes, including embryogenesis, vision, cell proliferation and differentiation, immune regulation, and glucose and lipid metabolism. Many of vitamin A’s functions are executed through retinoic acids that activate transcriptional networks controlled by retinoic acid receptors (RARs) and retinoid X receptors (RXRs).The liver plays a central role in vitamin A metabolism: (1) it produces bile supporting efficient intestinal absorption of fat-soluble nutrients like vitamin A; (2) it produces retinol binding protein 4 (RBP4) that distributes vitamin A, as retinol, to peripheral tissues; and (3) it harbors the largest body supply of vitamin A, mostly as retinyl esters, in hepatic stellate cells (HSCs). In times of inadequate dietary intake, the liver maintains stable circulating retinol levels of approximately 2 μmol/L, sufficient to provide the body with this vitamin for months. Liver diseases, in particular those leading to fibrosis and cirrhosis, are associated with impaired vitamin A homeostasis and may lead to vitamin A deficiency. Liver injury triggers HSCs to transdifferentiate to myofibroblasts that produce excessive amounts of extracellular matrix, leading to fibrosis. HSCs lose the retinyl ester stores in this process, ultimately leading to vitamin A deficiency. Non-alcoholic fatty liver disease (NAFLD) is the hepatic manifestation of metabolic syndrome and is a spectrum of conditions ranging from benign hepatic steatosis to non-alcoholic steatohepatitis (NASH); it may progress to cirrhosis and liver cancer. NASH is projected to be the main cause of liver failure in the near future. Retinoic acids are key regulators of glucose and lipid metabolism in the liver and adipose tissue, but it is unknown whether impaired vitamin A homeostasis contributes to or suppresses the development of NAFLD. A genetic variant of patatin-like phospholipase domain-containing 3 (PNPLA3-I148M) is the most prominent heritable factor associated with NAFLD. Interestingly, PNPLA3 harbors retinyl ester hydrolase activity and PNPLA3-I148M is associated with low serum retinol level, but enhanced retinyl esters in the liver of NAFLD patients. Low circulating retinol in NAFLD may therefore not reflect true “vitamin A deficiency”, but rather disturbed vitamin A metabolism. Here, we summarize current knowledge about vitamin A metabolism in NAFLD and its putative role in the progression of liver disease, as well as the therapeutic potential of vitamin A metabolites.

## 1. Vitamin A, Its Active Metabolites, and Dietary Causes of Vitamin A Deficiency

The generic term “Vitamin A” is used for compounds having the biological activity of retinol or its metabolic products, such as all-trans retinoic acid (atRA), 9-cis retinoic acid (9cRA), and 9-cis-13,14 dihydroretinoic acid (9cDHRA). Mammals cannot synthesize retinol themselves, so depend on the diet to acquire sufficient amounts of this micronutrient. Dietary sources of vitamin A are carotenoids, such as β-carotene (rich plant sources are sweet potatoes, carrots, and dark green leafy vegetables like spinach) and retinyl esters (rich animal sources are liver, eggs, and fish) [1]. Vitamin A is an essential fat-soluble vitamin and adequate daily intake (~700–900 μg for humans) and hepatic storage (~80% in a healthy individual) are required to maintain plasma retinol levels around 2 μmol/L in humans (1–2 μmol/L in rodents) [2,3]. Vitamin A plays important physiological roles in vision, reproduction, growth, development, immunity, and metabolic programs [4,5,6,7,8,9,10]. Most functions of vitamin A are mediated through the activation of ligand-activated transcription factors. For example, atRA is a high-affinity ligand for retinoic acid receptors (RARα, β and γ), while 9cRA and 9cDHRA activate retinoid X receptors (RXRα, β, and γ). The ligand-dependent activation and the physiological roles of RAR and RXR have been extensively reviewed in recent years [10,11,12].

Vitamin A deficiency (VAD) is common in low-income countries. According to the World Health Organization (WHO) an estimated 250 million preschool children in these countries have VAD and are predisposed to developmental and immune disorders, preventable (night) blindness, and infection-associated morbidity and mortality. Moreover, VAD in pregnant women increases the risk for maternal mortality, though high doses of vitamin A supplementation in pregnant women are contraindicated as they are associated with teratogenicity for the fetus [6,13]. The liver plays a central role in controlling vitamin A metabolism and chronic liver diseases, like biliary atresia [14,15], primary biliary cholangitis (PBC) [16,17,18], primary sclerosing cholangitis (PSC) [19], viral hepatitis [20], alcoholic liver disease (ALD) [21,22], and non-alcoholic fatty liver disease (NAFLD, NASH) [23,24,25] are associated with VAD. Moreover, VAD in hepatitis C virus (HCV)-infected patients is associated with a lack of response to interferon-based antiviral therapy [20]. Thus, VAD is also a highly relevant condition in high-income countries given the increased incidence of liver diseases associated with a Western lifestyle, e.g., 20–30% of the population of industrialized countries is suspected of having NAFLD [26,27,28]. Many epidemiological studies have reported VAD in liver diseases, including NAFLD, where the golden standard for assessing the vitamin A status is measuring serum retinol levels (<0.7 μmol/L is considered VAD) [29]. Moreover, serum retinol levels are inversely associated with disease progression [11,24,25,30,31]. Oral vitamin A supplements are often not very effective in restoring adequate serum retinol levels in NAFLD patients, indicating a fundamentally impaired vitamin A metabolism. Animal studies have shown the beneficial effects of therapeutic use of vitamin A derivatives, e.g., retinoic acids or pharmacological modulators of RARs and RXRs. Relatively little is known, however, about changes in vitamin A metabolism in liver disease, including possible changes in storage (retinyl esters) vs. circulating (retinol, RA) forms and possible redirection of vitamin A pools within the liver (hepatocytes vs. hepatic stellate cells) and/or to extrahepatic tissues. Insight into these processes is needed to get a better view of the possible therapeutic application of vitamin A and/or its derivatives (retinoids acting on RARs and rexinoids acting on RXRs) in liver diseases. This review aims to give an overview of vitamin A metabolism in patients and animal models of NAFLD and associated obesity, as well as the possible therapeutic value of vitamin A and its metabolites in NAFLD.

## 2. Vitamin A Uptake, Storage, and Metabolism

**Figure 1:**
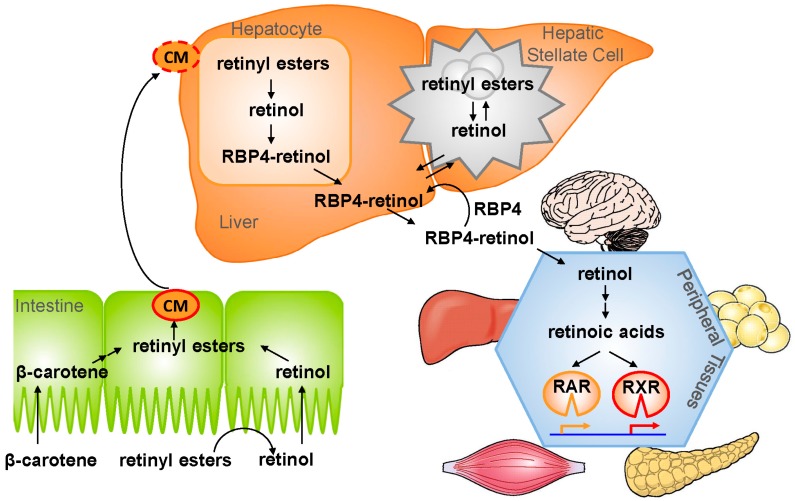
Schematic representation of intestinal vitamin A absorption, transport to, and storage in the liver and redistribution to peripheral tissues (see main text for details). Carotenes (from plants) and retinyl esters (from animals) are the main dietary sources of vitamin A (lower left corner). They are absorbed in the proximal small intestine and transported as retinyl esters in chylomicrons (CM) to the liver. Chylomicron remnants are taken up by hepatocytes and retinyl esters are hydrolyzed to form retinol. Hepatocytes produce retinol binding protein 4 (RBP4) and retinol binding to RBP4 stimulates the secretion of retinol-carrying holo-RBP4 to the circulation. Retinol is then either transported to hepatic stellate cells for storage as retinyl esters or transported to peripheral tissues where it is converted to retinoic acids that activate the transcription factors retinoic acid receptor (RAR) or retinoid X receptor (RXR). In times of inadequate vitamin A intake, retinol is released from the HSC stores to maintain stable levels of circulating retinol (~2 μM in human).

A schematic representation of vitamin A uptake, transport, storage, and metabolism is given in Figure 1. Plant-derived carotenes and animal-derived retinyl esters are the main sources of vitamin A in the diet. Bile salts produced by the liver are important to keep these fat-soluble compounds in solution in the digestive tract and make them available for absorption in the proximal part of the small intestine [32,33]. Retinyl ester hydrolases (REH) in the gut lumen release retinol from retinyl esters, after which it is absorbed by enterocytes by a yet to-be-characterized mechanism. Carotenes are taken up by membrane-bound transporters, including fatty acid translocase (FAT/CD36), Niemann–Pick C1-Like 1 (NPC1L1) and scavenger receptor class B member 1 (SR-B1), and metabolized to retinol inside enterocytes. Next, retinol is re-esterified to retinyl esters, mainly by lecithin:retinol acyl transferase (LRAT) and diacylglycerol *O*-acyltransferase (DGAT1), sequestered into chylomicrons (CM) and secreted to the circulation [34,35]. CM remnants that still contain most of the retinyl esters are taken up by hepatocytes, mostly through the LDL receptor (LDLR) [36]. The retinyl esters are then hydrolyzed to retinol through the action of REHs [37], followed by transfer of retinol to RBP4 and transthyretin (TTR), which stimulates its secretion into the circulation [38,39,40]. From here, retinol is directed to either of two main destinations: (1) peripheral tissues in demand of retinol (-metabolites). Here, the RBP4-TTR-retinol complex binds to “stimulated by retinoic acid gene 6 homolog” (STRA6), which facilitates uptake of retinol [41] and makes it available for conversion to bioactive retinoic acids; or (2) hepatic stellate cells (HSC) that convert it back again to retinyl esters (by LRAT and DGAT1 [42,43]) and store it in large cytoplasmic lipid droplets. It is estimated that 60–95% of the whole body’s reservoir of vitamin A resides in the liver of a healthy individual, but considerable amounts may also reside in adipose tissue, the pancreas, the intestines, and the eyes. Still, the liver is considered to be the central player in providing retinol to peripheral tissues in times of insufficient dietary intake. This is a tightly controlled process maintaining well-balanced levels of approximately 2 μmol/L retinol in the blood. However, few details of how this is achieved are known, e.g., it is unknown how retinol gets in and out of HSC and which (molecular) signals control this. Mobilization of retinol from lipid droplets in HSC is catalyzed by REHs, and several enzymes are implicated in this process, including adipose triglyceride lipase (ATGL/PNPLA2) [44], patatin-like phospholipase domain-containing 3 (PNPLA3) [44,45,46], and hormone-sensitive lipase (HSL) [47,48]. Interestingly, genetic studies have identified the PNPLA3-I148M variant as a prominent genetic factor associated with NAFLD, and even more prominently with disease progression within NAFLD to NASH and NASH-associated cirrhosis [49,50,51]. PNPLA3 affects vitamin A homeostasis, as will be discussed in more detail in the following sections. Liver diseases, in particular chronic liver diseases that lead to liver fibrosis, have major impact on hepatic vitamin A metabolism and storage. Quiescent vitamin A-storing HSCs (qHSCs) become activated as a result of liver injury, and transdifferentiate to migratory and highly-proliferative myofibroblasts (aHSCs) that produce excessive amounts of extracellular matrix proteins (ECM), mainly collagens and fibronectins, leading to fibrosis. HSCs lose their vitamin A stores during this transdifferentiation process. Consequently, chronic liver diseases, including NAFLD, may lead to vitamin A inadequacy.

Given the various forms and storage sites of vitamin A in the body, it is unclear whether the reported vitamin A deficiency (VAD) in NAFLD is truly a reflection of complete depletion of vitamin A from the body, or whether it is rather a reflection of impaired vitamin A metabolism. Clinical examination of vitamin A status is typically performed by determining serum retinol levels, where levels below 0.7 μmol/L are considered deficient. Retinol is also present in the liver, but most vitamin A is esterified, predominantly as retinyl palmitate. Both forms reside in hepatocytes (the initial site of absorption from the circulation) and HSCs (the final destination and controller of serum retinol levels). In addition, a significant amount of vitamin A is present in adipose tissue and the pancreas, both as retinol and retinyl esters. Thus, for thorough evaluation of the vitamin A status, both retinol and retinyl esters need to be quantified in the blood, the liver, and preferably also the adipose tissue. Hepatic retinol levels are sometimes analyzed in liver biopsies or explant livers [25,52], but quantification of retinyl ester is rarely performed [52]. Therefore, when scanning the literature for the vitamin A status in liver disease, it is important to take into account what exactly is measured and keep in mind that the limited data on hepatic retinyl ester levels prevents us from establishing the true VAD prevalence in humans.

## 3. Vitamin A and RBP4 in the Clinical Course of NAFLD and Metabolic Syndrome

NAFLD is characterized by the accumulation of fat in the liver, in particular non-esterified fatty acids (NEFA), triglycerides, and non-esterified cholesterol. NAFLD is regarded as a consequence of excessive dietary intake of fat and/or sugars (glucose and fructose) typical of the “Western” lifestyle, in combination with limited physical activity. Benign steatosis may progress to an inflammatory state in the liver, leading to chronic liver injury, e.g., non-alcoholic steatohepatitis (NASH). In turn, this causes a chronic healing response leading to liver fibrosis, which may progress to cirrhosis, which predisposes for liver cancer. NAFLD is commonly appreciated to be the hepatic manifestation of metabolic syndrome (MetS). Numerous studies have reported on vitamin A status in MetS, while reports specifically focusing on NAFLD are limited. Almost all studies documenting vitamin A status in MetS report reductions in serum retinol, retinoic acid, and/or β-carotene that are inversely correlated with MetS features, including obesity, insulin resistance, glucose intolerance, and hypertriglyceridemia [53,54,55,56,57,58,59,60,61,62]. In line with these observations, inadequate serum retinol levels (<1.05 μmol/L) were found in 11–36% of morbidly obese adults with ultrasonography-proven NAFLD, and a significant association between low retinol levels and insulin resistance (IR) was found [25,53]. A similar trend was observed in obese children with NAFLD [23]. Moreover, serum retinol levels were inversely associated with body mass and serum transaminases in patients with NAFLD, suggesting a link between retinol inadequacy and development of disease [24]. In one study, both serum and hepatic retinol levels were analyzed in NAFLD patients and the latter was more frequently found to be inadequate (36% vs. 68% for serum and hepatic retinol inadequacy, respectively) in these patients [25]. Hepatic retinol levels showed a strong inverse correlation with the histological classification of the disease (sub-classified in mild and severe steatosis, NASH and hepatocyte necrosis), which was not observed for serum retinol levels. These observations were confirmed by Trasino et al. [63], who reported an inverse correlation between the level of steatosis and hepatic retinol and retinyl palmitate concentrations. However, the cause of hepatic steatosis was unknown in this study, as liver tissue was obtained from deceased individuals after motor vehicle accidents or head trauma [63]. More recently, serum retinoic acid levels were also shown to be inversely associated with hepatic steatosis and liver injury in NAFLD. While reference values for retinol are approximately 2 μmol/L in human blood, atRA concentrations are approximately 200-fold lower (~10 pmol/L). Still, also for circulating atRA, levels were markedly reduced in NAFLD (−47%) and even more pronounced in NASH (−58%) patients compared to control subjects [64]. Moreover, atRA concentration and RXRα levels were inversely correlated to liver triglyceride content, grade of hepatic steatosis, and severity of liver disease [64]. In comparing the expression of 51 genes involved in RA signaling in control, simple steatotic, and NASH livers, Ashla et al. [65] observed a hyper-dynamic state of RA metabolism and degradation in the liver of NAFLD patients, which further increased when it included NASH. Hepatic expression of genes involved in vitamin A storage (LRAT and DGAT1) as well as RA production retinaldehyde dehydrogenase 1 and 3 (RALDH1 and 3) and degradation (Cyp26A) were all significantly increased in NAFLD patients. This expression profile may indeed lead to low hepatic retinol and retinoic acids levels, but not necessarily to VAD, as it suggests that production of retinyl esters is also enhanced. Low retinol levels appear predictive for the development of hepatocellular carcinoma (HCC) in cirrhotic patients [66,67], though this has not yet been studied specifically for NAFLD-associated HCC. Impaired RAR- and RXR-mediated signaling is assumed to promote HCC. Hepatocyte-specific overexpression of a dominant negative form of RAR induces hepatic tumor development, which is suppressed by a diet containing retinoic acid [68].

In contrast to reduced retinol, serum levels of RBP4 are typically elevated in MetS patients and obese animals. A landmark paper by Yang et al. [69] revealed a direct role of serum RBP4 in the development of insulin resistance in obesity and type 2 diabetes [69]. RBP4 expression was selectively enhanced in adipose tissue in animal models of type 2 diabetes, but not in the liver. Transgenic overexpression of human RBP4 in mice or injection with recombinant RBP4 in normal mice led to insulin resistance. These findings suggest that adipose-derived RBP4 increases serum levels of RBP4 and plays a pathological role in type 2 diabetes (T2D). Many papers have confirmed the elevated serum RBP4 levels in obese patients with or without T2D (for example see [69,70,71,72,73]). However, a significant number of similar studies did not replicate the enhanced levels of RBP4 in the serum of these patients (for example see [74,75,76,77,78]). Thus, the specific correlation between serum RBP4 levels and components of the MetS spectrum remains an active debate today. Since serum retinol levels are typically reduced in MetS, this feature may be an important factor to consider together with RBP4. Low serum retinol in combination with stable or enhanced RBP4 levels implies the presence of more retinol-free (apo-) RBP4 in circulation. The few studies that quantified both retinol and RBP4 in serum indeed confirm that a low retinol-to-RBP4 ratio is a better predictor for obesity, T2D, and other components of metabolic syndrome in children and adults then RBP4 alone [79,80,81].

In addition to the debate on the relevance of serum RBP4 levels as indicator for MetS components, the origin of the increased levels of RBP4 is also an ongoing puzzle. Enhanced RBP4 production by adipose tissue was the original hypothesis, because of the selective increase of RBP4 mRNA levels in this tissue in T2D mice but not in the liver [69]. More recent data indicate that elevated serum RBP4 levels are specifically associated with obesity- and T2D-associated reduced kidney function, suggesting that impaired renal clearance of RBP4 is an important contributing factor [82,83,84,85,86]. Recently, it was found that hepatocyte-specific deletion of *Rbp4* in mice completely abolishes RBP4 from the circulation, both in lean and obese animals, providing strong evidence that the liver, more specifically the hepatocytes, is the primary—if not sole—source of serum RBP4 [87]. In line with this finding is that adipocyte-specific overexpression of human RBP4 did not increase circulating RBP4, but did cause hepatic steatosis in mice [88]. These findings suggest that adipocyte-produced RBP4 acts locally to activate signaling cascades that cause fat accumulation in the liver, but it is not a circulating adipokine itself. Secretion of RBP4 by hepatocytes is strongly stimulated by retinol [38,39,40]; this may be impaired in NAFLD patients when both hepatic and serum retinol levels are reduced. RBP4 indeed accumulates in the livers of NAFLD patients, as determined by immunohistochemistry [89]. Hepatic RBP4 retention in low-retinol NAFLD livers suggests that impaired renal clearance might even be a more prominent factor in enhancing serum RBP4 levels.

Bariatric surgery is nowadays a common approach to treat morbid obesity, where an adjustable gastric band (AGB), Roux-en-Y gastric bypass (RYGB), biliopancreatic diversion with a duodenal switch (BPD-DS), and vertical sleeve gastrectomy (VSG) are the four most common procedures. Remarkably, serum retinol levels do not return to normal after bariatric surgery and in most studies even further decline in the 6–12 months post-surgery, also under impressive weight loss and improvement of MetS components [90,91,92,93,94]. Ocular problems related to low vitamin A status, such as night blindness, are commonly reported in patients who underwent bariatric surgery. BPD-DS appears to induce a stronger decline in serum retinol compared to RYGB [91,95] and vitamin A deficiency was still observed in 23–69% of patients 4–10 years post-PBD-DS [96,97,98]. Impaired serum retinol levels were also observed in neonates from mothers who underwent RYGB 11–69 months before the onset of pregnancy, which may cause irreversible eye problems in the child [99]. In contrast to retinol, circulating levels of vitamin D and E were higher in neonates of mothers who had undergone bariatric surgery as compared to neonates from healthy mothers [100]. This argues against a general deficiency in fat-soluble vitamins in these children. Inadequate serum retinol levels before and/or after bariatric surgery are often linked to inadequate dietary intake, as well as the anatomical changes resulting from bariatric surgery. Large parts of the jejunum do not receive dietary input post-surgery, and this is exactly the site where most vitamin A is normally absorbed. However, intramuscular [101] or dietary vitamin A supplementation (alone or in multivitamins) does not effectively elevate serum retinol levels and/or prevent the decline in serum retinol post-surgery [91,92,94,102]. A few case reports have shown that only very high doses of vitamin A relieve the signs of severe VAD, like night blindness [103]. Little is currently known about the effect of diet-induced weight loss on serum retinol levels. Serum RBP4 declines after this treatment, but this is not consistently associated with an improvement in insulin sensitivity [104,105,106,107].

## 4. PNPLA3 Variant I148M Regulates Vitamin A in NAFLD

Several genomic loci have been identified to increase the susceptibility for NAFLD. The most prominent NAFLD-associated genetic risk factor is a specific variant of PNPLA3, PNPLA3-I148M, which also predisposes for disease progression and NAFLD-associated hepatocellular carcinoma [108,109,110,111]. PNPLA3 is a multifunctional enzyme acting as a triglyceride hydrolase, an acetyl-CoA-independent transacylase, and a retinyl esterase [112]. Originally, it was found to contain hydrolase activity towards triglycerides, in particular those containing mono- and poly-unsaturated fatty acids. PNPLA3-I148M shows reduced hydrolase activity and promotes hepatic triglyceride accumulation, all in line with the primary phenotype of NAFLD: fat accumulation in hepatocytes. Likewise, chronic overexpression of PNPLA3-I148M (but not PNPLA3-I148) in mice leads to hepatic steatosis [113]. Notably, while the variant is associated with increased liver fat content, PNPLA3-I148M appears not to be associated with other features of metabolic syndrome, like insulin resistance [114,115]. Serum triglycerides (TG) levels are either the same or lower in PNPLA3-I148M carriers compared to non-carriers, consistent with a lack of association with insulin resistance [115].

A recent paper, however, showed that PNPLA3 mRNA and protein levels are significantly higher in HSC compared to hepatocytes. PNPLA3 was found to contain retinyl esterase activity and promotes the release of retinol from lipid droplets [45]. Carriers of the PNPLA3-I148M allele with either NAFLD or obesity alone have reduced fasting circulating retinol and RBP4 levels. No association was found between this genotype and β-carotene, indicating a specific association with retinol [46]. On the other hand, hepatic retinyl palmitate levels are increased in individuals homozygous for PNPLA3-I148M [116], supporting a role for PNPLA3 in hepatic retinoid metabolism [116]. PNPLA3 expression in HSC is regulated by retinol and insulin and is inversely related to lipid droplet content. Retinol suppresses PNPLA3 expression in HSC, while it is induced upon retinol depletion. Moreover, PNPLA3 expression is induced upon HSC activation and the PNPLA3-I148M variant further promotes fibrogenic features of HSC, including enhanced proliferation, migration and expression of collagen type 1 alpha 1 (COL1A1), pro-inflammatory cytokines, and chemokines alongside lower cellular retinol levels. Remarkably, PNPLA3-I148M-carrying HSC contain more lipid droplets, which is a typical characteristic of HSC quiescence [117]. These features are in line with a higher risk for progressive liver disease in PNPLA3-I148M carriers, but seemingly in contrast to the increased hepatic retinyl palmitate levels in those patients. Thus, these NAFLD patients did not have VAD, although the low circulating retinol levels are interpreted as such. It remains to be determined whether this is a more general phenomenon in NAFLD. Since (1) dietary retinyl esters are first delivered to—and hydrolyzed—in hepatocytes before they move as retinol to HSC to become esterified again, and since (2) PNPLA3 is expressed both in hepatocytes and HSC, it remains unclear which hepatic cell type retinyl esters accumulate in NAFLD and specifically in PNPLA3-I148M patients.

## 5. Vitamin A and Hepatic Lipid Metabolism

**Figure 2:**
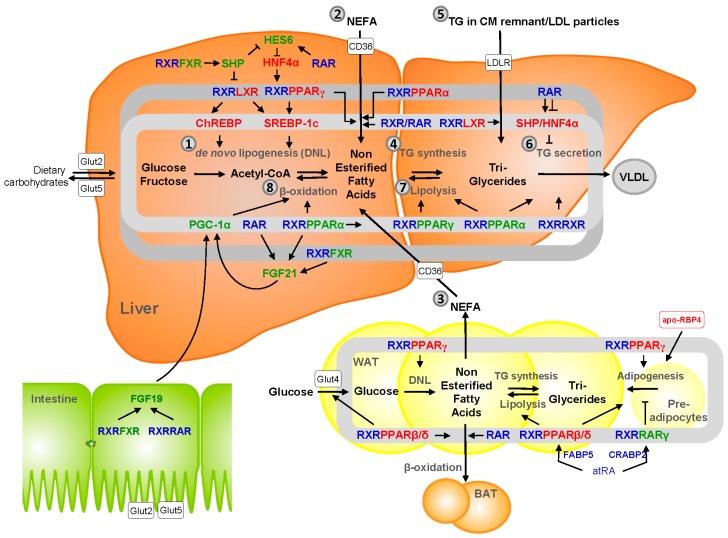
Regulation of hepatic lipid metabolism by vitamin A metabolites. Triglyceride synthesis and breakdown is subdivided into eight steps: (1) de novo lipogenesis (DNL) in the liver, (2) influx of dietary lipids (delivered as non-esterified free fatty acids (NEFAs) or as triglycerides (TG) in chylomicrons), (3) influx of NEFAs produced by adipose tissue (primarily from white adipose tissue (WAT)), (4) esterification of lipids (mainly to TG) and packaging into lipid droplets, (5) influx of TG carried in CM remnants and low density lipoproteins (LDL), (6) efflux of TG carried in very low density lipoprotein (VLDL)-particles, (7) TG hydrolysis producing NEFAs, and (8) catabolism of NEFAs through mitochondrial and peroxisomal β-oxidation. Direct transcriptional regulation of lipogenic/lipolytic genes is shown in the inner (light gray) ring. Indirect transcriptional regulation is shown inside or outside the outer (dark gray) ring. Vitamin A-related factors are indicated in blue. Factors that promote lipogenesis are shown in red; factors promoting lipolysis in green. Relevant regulation and factors in adipose tissue and the intestine are also included (see main text for details about the specific genes that are regulated in each step). Additional abbreviations: FXR—Farnesoid X Receptor, SHP—Small Heterodimer Partner 1, HES6—Hes family BHLH transcription factor 6, HNF4α—Hepatocyte Nuclear Factor 4 alpha, LXR—Liver X Receptor, PPARγ—Peroxisome Proliferator-Activated Receptor gamma, chREBP—carbohydrate Response Element Binding Protein, SREBP-1c—Sterol Response Element Binding Protein-1c, PGC-1α—PPARγ-Coactivator 1-alpha, FGF—Fibroblast Growth Factor, BAT—Brown Adipose Tissue.

Hepatic lipid content is a result of the following steps: (1) de novo lipogenesis (DNL) in the liver; (2) an influx of dietary lipids (delivered as non-esterified free fatty acids (NEFAs) or as TG in chylomicrons); (3) an influx of NEFAs produced by adipose tissue (primarily from white adipose tissue (WAT)); (4) the esterification of lipids (mainly to TG) and packaging into lipid droplets; (5) an influx of TG carried in CM remnants and low density lipoproteins (LDL); (6) an efflux of TG carried in very low density lipoprotein (VLDL)-particles; (7) TG hydrolysis producing NEFAs; and (8) catabolism of NEFAs through mitochondrial and peroxisomal β-oxidation (Figure 2). NAFLD is characterized by hepatic TG accumulation, enhanced VLDL production, and secretion leading to hypertriglyceridemia. Vitamin A metabolites, especially retinoic acids, are involved in regulatory networks that affect all these processes either directly or indirectly, as described below and schematically depicted in Figure 2.

Hepatic DNL (#1 in Figure 2) is under primary the transcriptional control of the sterol response element binding protein-1c (SREBP-1c) and carbohydrate response element binding protein (ChREBP), which induce expression of key glycolytic enzymes (glucokinase (GCK), pyruvate kinase isozymes R/L (PKLR), ATP citrate lyase (ACL), acetyl-CoA synthetase (ACS) and lipogenic enzymes (acetyl-CoA carboxylase 1 (ACC1), fatty acid synthase (FASN), ELOVL fatty acid elongase 6 (ELOVL6), stearoyl-CoA desaturase-1 (SCD), glycerol-3-phosphate acyltransferase, mitochondrial (GPAT)) in the liver. The absence or inhibition of SREBP-1c or ChREBP impairs lipid synthesis and reduces hepatic steatosis [118,119,120]. Insulin, glucose, and fructose stimulate SREBP-1c and ChREBP expression to promote hepatic DNL. Retinoic acids, as well as synthetic ligands of RXRα (e.g., bexarotene), enhance hepatic DNL and plasma TG levels by activating Liver X receptor (LXR)/RXR and peroxisome proliferator-activated receptor γ (PPARγ)/RXR, which, in turn, enhance expression of SREBP-1c and ChREBP [121]. Although LXR and PPARγ are typically activated by oxycholesterols and NEFAs, respectively, RXRα is a permissive dimerization partner, meaning that RXR ligands enhance DNL through those heterodimers independently of the co-presence of ligands for LXR or PPARγ. LXR/RXR also directly induces FAS expression, thereby promoting DNL and enhancing plasma triglyceride levels in mice [122]. In contrast, atRA suppresses DNL by activating RARα, which, via induction of Hes family BHLH transcription factor 6 (HES6) and subsequent inhibition of hepatocyte nuclear factor 4α (HNF4α), reduces PPARγ expression and downstream SREBP-1c activity. In a counteracting mechanism, 9cRA-activated RXRα induces expression of a small heterodimer partner (SHP), which inhibits HES6 expression, promoting DNL via the PPARγ-SREBP-1c axis [123]. However, SHP also inhibits LXR/RXR transcriptional activity, thereby simultaneously inhibiting SREBP-1c-mediated DNL [124]. This highlights the delicate position of SHP in the development of hepatic steatosis, inhibiting RXR/LXR-ChREBP/SREBP-1c-mediated DNL, while at the same time promoting DNL via the HES6-HNF4α-PPARγ pathway. The absence of SHP protects mice from diet-induced hepatic steatosis, suggesting a most prominent role for the HES6-HNF4α-PPARγ axis, which is activated by atRA and RARα [123].

Influx of NEFA, either from dietary sources or adipose tissue (#2, 3 in Figure 2) is facilitated by fatty acid translocase (FAT/CD36). Hepatocyte-specific deletion of CD36 protects against HFD-induced lipid accumulation in the mouse liver, underscoring its role in NAFLD pathogenesis. Expression of CD36 is controlled by SREBP-1c, RXR/PPARγ, and RXR/PPARα, transcription factors that are regulated directly or indirectly by RA (see above). Moreover, RAR/RXR may also directly enhance expression of CD36, though this regulatory pathway has only been studied in human THP-1 monocytes so far [125,126].

Hepatic TG synthesis (#4 in Figure 2) is catalyzed by GPAT, mannosyl (alpha-1,6-)-glycoprotein beta-1,2-*N*-acetylglucosaminyltransferase (MGAT2), and diacylglycerol *O*-acyltransferase 2 (DGAT2), all of which are under the transcriptional control of ChREBP. In addition, GPAT is controlled by SREBP-1c. Though no specific data are available on the RA-mediated expression of those genes, it is likely that they are co-regulated with key genes in DNL as a result of RXR/LXR and RAR-mediated effects on ChREBP and SREBP-1c.

Hepatic uptake of TG-containing CM remnants or LDL particles (#5 in Figure 2) is controlled by the LDL receptor (LDLR). Expression of human LDLR is controlled by LXR/RXR [127], but co-regulation by retinoic acids has not yet been studied in detail. However, given the potent effects of RXR ligands on LXR/RXR-mediated regulation of SREBP-1c and ChREBP (described above), it is likely that retinoic acids may also promote the LDLR-mediated uptake of TG in the liver.

Hepatic VLDL particle formation and secretion (#6 in Figure 2) are facilitated by apo-CIII [128]. Apo-CIII null mice fail to stimulate VLDL production upon HFD feeding, while Apo-CIII overexpressing mice show enhanced diet-induced triglyceride accumulation in the liver [129]. A genetic variant of Apo-CIII leads to enhanced circulating Apo-CIII in humans and is associated with NAFLD [130]. Hepatic Apo-CIII expression is suppressed by RARα via a pathway involving SHP and HNF4α, thereby reducing hepatic and plasma triglyceride levels [131]. Earlier studies have shown that RXR ligands sort the opposite effect and enhance hepatic apo-CIII expression, either via RXR homodimers or RXR/PPARα, and thereby promote hypertriglyceridemia, a well-known adverse effect of pharmacological ligands of RXR and a risk factor for cardiovascular disease (CVD) [132]. This emphasizes the opposite roles of RAR ligands (e.g., atRA) and RXR-ligands (e.g., 9cRA) on VLDL particle production and secretion by the liver. In addition, Apo-CIII expression is controlled by ChREBP, adding an additional layer of indirect RA responsiveness, as described above [133].

Key enzymes involved in TG lipolysis (#7 in Figure 2) in hepatocytes are adipose triglyceride lipase (ATGL/PNPLA2), hormone-sensitive lipase (HSL), and PNPLA3. Those are exactly the same enzymes responsible for retinyl ester hydrolysis that release retinol from cellular stores, though that activity is believed to occur predominantly in HSC (see above). The absence of either ATGL or HSL aggravates diet-induced hepatic TG accumulation and steatosis in mice [134,135,136]. Hepatic overexpression of ATGL and/or HSL reduced TG levels by 40–60% in ob/ob mice and enhanced fatty acid oxidation and ameliorated hepatic steatosis, while fasting plasma TG and NEFA were not affected [137]. The expression of both ATGL and HSL is controlled by PPARα, and the PPARα-agonist-mediated decrease in hepatic lipids in HFD-fed mice was mirrored by a strong increase in the expression of these genes [138]. PPARα ligands also induce hepatic ATGL expression in rats and reduce hepatic TG levels [139]. It is interesting to note that hepatic lipolysis produces ligands for PPARα to further enhance catabolism via mitochondrial and/or peroxisomal β-oxidation. Moreover, HSL expression is under the (positive) control of PPARγ [140]. LXRα-agonists, on the other hand, were found to suppress HSL expression but, so far, this has only been analyzed in adipocytes [141]. There is no information available on the effect of RA on PPARα- and/or PPARγ-mediated regulation of ATGL and HSL, but these vitamin A metabolites are likely have modulatory functions on their expression given the collaborative actions of those factors with RXRα. Interestingly, PNPLA3 expression is under the direct control of SREBP-1c and ChREBP [142,143], which was linked to its role in the conversion of TG to NEFA. Given recent data that PNPLA3 also contains retinyl esterase activity and is highly expressed in HSC, this may imply a direct role of these transcription factors in vitamin A metabolism.

Finally, hepatic fatty acid β-oxidation (#8 in Figure 2) is largely controlled by PPARα/RXR. There is a wealth of information about PPARα agonists and how they protect against and/or relieve fat accumulation in the liver (for recent reviews, see [144,145]). There is, however, limited information on whether vitamin A-metabolites have a modulatory effect on RXR/PPARα-mediated fatty acid catabolism. atRA treatment does enhance hepatic PPARα and RXRα levels in mice, as well as key target genes uncoupling-protein-2 (UCP2), carnitine-palmitoyltransferase 1A (CPT1), and carnitine/acylcarnitine carrier, while suppressing SREBP-1c and FAS levels [146]. Both 9cRA and atRA were shown to induce CPT1 in vitro, most likely via RXR/PPARα [147]. PPARα also induces expression of Fibroblast Growth Factor 21 (FGF21), a hepatocyte-derived hormone suppressing obesity-induced fatty liver. FGF21 controls glucose and lipid metabolism and induces PPARγ-coactivator 1-alpha (PGC-1α) signaling, resulting in enhanced fatty acid oxidation and suppression of lipid synthesis (reviewed in [148]). atRA induces expression of FGF21 through RARα and RARβ. Adenoviral overexpression of RARβ enhances hepatic production and secretion of FGF21 and promotes hepatic fatty acid β-oxidation [149]. FGF21 expression is also controlled by RXR/Farnesoid X receptor (FXR), where RXR acts as a permissive partner. Thus, FGF21 expression is enhanced by 9cRA via FXR/RXR [150]. Hepatic lipid metabolism is also modulated by another FGF, e.g., FGF19 (the ortholog of FGF15 in rodents). Murine FGF15 is produced in the intestine, while human FGF19 is produced in the intestine as well as in the liver [151,152,153]. FGF19 suppresses lipogenesis by blocking SREBP-1c signaling and simultaneously inducing fatty acid β-oxidation by blocking ACC2 (reviewed in [154]). Mouse FGF15 expression is under the control of RXR/FXR, where ligands of RXR induce its expression independently from bile acid-induced FXR activation. A similar effect of retinoic acids was found for human FGF19 expression; however, mechanistically, a more prominent role was observed for RXR/RAR-mediated regulation of FGF19 [155].

Taken together, it is evident that vitamin A metabolites are key (co-)regulators of hepatic lipid metabolism. RAR-mediated signaling (via atRA or synthetic ligands) most consistently induces suppression of hepatic NEFA and TG accumulation. RXR-mediated signaling (via 9cRA, synthetic ligands, or other NR, like PPARs, LXR, and FXR) may cause opposing effects at different levels in the metabolic pathway, leading to hepatic lipid accumulation. However, the liver is not the only tissue involved in obesity-induced hepatic steatosis that is heavily regulated by vitamin A metabolites. The most important—and most intensively studied—being adipose tissue, which is discussed next.

## 6. Vitamin A and Fat Metabolism in Adipose Tissue

Besides controlling lipid metabolism in the liver, vitamin A metabolites also play key roles in the differentiation, maturation, and function of adipose tissue. In obesity-associated NAFLD, there is an increase in NEFA flowing from adipose tissue to the liver, in part as a result of insulin resistance. Adipogenesis is a tightly regulated cellular differentiation process, in which preadipocytes are transformed into lipid-storing adipocytes with enhanced expression of lipogenic genes. Insulin promotes glucose transport to adipocytes and PPARγ-dependent DNL leads to lipid accumulation.

atRA has a dual role in adipocyte differentiation and functionality: (1) it suppresses adipogenesis and (2) promotes lipolysis in differentiated adipocytes. Two RA-binding proteins, cellular retinoic acid binding protein 2 (CRABP2) and Fatty Acid Binding Protein 5 (FABP5), play a key role in directing atRA to either RXR/RARγ or RXR/PPARβ/δ, activation of which differentiates between the two pathways. atRA inhibits adipogenesis through the CRABP2-RXR/RARγ pathway, which induces expression of inhibitors of adipocyte differentiation, including SOX9, which blocks CCAAT/enhancer binding protein beta (C/EBPβ) and CCAAT/enhancer binding protein gamma (C/EBPγ)-mediated differentiation, and Kruppel-like factor 2 (KLF2), which blocks CCAAT/enhancer binding protein alpha (C/EBPα)-, SCREBP-1c-, and PPARγ-mediated adipogenesis. KLF2 also induces RARγ and CRABP2, providing a positive feedback loop to suppress adipogenesis [156,157]. Adipocyte differentiation (for instance, induced by insulin) is initiated by lowering CRABP2 levels and redirection of atRA-mediated signaling to FABP5-RXR/PPARβ/δ, which induces lipolysis (via HSL upregulation), mitochondrial activity (via uncoupling proteins/UCP1), and fatty acid β-oxidation, as well as enhancing the insulin-responsive glucose transporter type 4 (GLUT4). In vivo, atRA raises body temperature, decreases body weight, and reduces plasma triglycerides and insulin levels in obese mice, and might do so more potently than selective PPARβ/δ ligands [156,157]. Obviously, this is not a stand-alone effect on adipose tissue, but heavily intertwined with the effects of hepatic lipid metabolism described above. Interestingly, impaired supply of retinols to (pre)adipocytes may also stimulate adipogenesis, as an excess of (retinol-free) apoRBP4 (as observed in obese individuals with a low retinol:RBP4 ratio) promotes retinol efflux via STRA6, thereby reducing RAR activity and leading to enhanced adipogenesis [158]. As stated earlier, adipocytes are the main extrahepatic cells that express RBP4, but this does not contribute to circulating RBP4. Adipocyte-specific overexpression of (human) RBP4 aggravated diet-induced obesity, glucose intolerance, and hepatic TG levels. RBP4-induced inflammation in adipose tissue stimulated lipolysis in adipocytes, leading to enhanced circulating NEFA, leading to elevated triglycerides in the liver [88].

In addition to atRA signaling, synthetic ligands for RXR promote adipogenesis in 3T3-L1 cells, a commonly used model to study adipocyte differentiation, by activation of RXR/PPARγ-mediated adipogenesis [159]. On the other hand, retinaldehyde, the precursor for retinoic acid, inhibits 9cRA-mediated activation of RXR/PPARγ thereby suppressing adipogenesis and lipid accumulation in adipose tissue. Moreover, retinaldehyde activates RAR, thereby recruiting PGC-1α and inducing UCP1 expression leading to enhanced mitochondrial respiration and adaptive thermogenesis, which promotes “browning” of white adipose tissue [160]. While endogenous levels of retinoic acids are generally undetectable in tissues, retinaldehyde levels in mouse adipose tissue are ~1 nmol/g [161]. Intraperitoneal administration of retinaldehyde significantly suppressed adipogenesis and diet-induced obesity in mice, while such an effect was not observed after oral delivery of retinaldehyde [161,162]. Genetic ablation of *Aldh1a1* (encoding RALDH1) and administration of RALDH inhibitors increase tissue levels of retinaldehyde and protect against diet-induced obesity and diabetes [160,161].

Finally, glucose may be an important modulator of the effect of RA on adipocytes as recent data show that RA suppresses lipid accumulation under normal glucose levels, while this effect shifts to lipid accumulation at high glucose conditions, a metabolic switch controlled by SREBP-1c [163].

## 7. Insulin and Vitamin A Cross-Talk in NAFLD

Vitamin A is required for normal development and endocrine functions of the pancreas, including the insulin-producing β-cells and the glucagon-producing α-cells in the islets of Langerhans. The pancreas stores retinoids in pancreatic stellate cells that are essential for normal islet function. Similar to the liver, retinoid storage in the pancreas is impaired during development of pancreatic diseases. VAD reduces β-cell mass and increases α-cell mass. Consequently, VAD in mice leads to aberrant pancreatic endocrine function due to lower insulin secretion and promotes hyperglycemia [164]. atRA-activated RARα induces pancreatic glucose transporter type 2 (Glut2) and Gck expression and is required in the adult pancreas for maintaining β-cell mass and function [165,166]. Similarly, RARβ2 agonists improved insulin sensitivity, lowered serum glucose and insulin levels, and reduced triglycerides and steatosis in the liver, pancreas, and kidneys of obese and diabetic mice [167].

In the liver, insulin promotes the activation of HSC via the phosphorylation of forkhead box gene, group O1 (FoxO1). Active (non-phosphorylated) FoxO1 suppresses HSC activation. Insulin signals via the PI3/AKT pathway to phosphorylate FoxO1, thereby allowing activation of HSC, characterized by enhanced proliferation and expression of fibrotic markers and aggravation of bile duct ligation-induced fibrosis in FoxO1+/− heterozygous mice compared to FoxO1+/+ wild types [168,169,170]. A recent paper suggests, though, that the effect of insulin may be reversed in the presence of vitamin A. Co-treatment with insulin potentiated the vitamin A-mediated suppression of HSC activation markers through stimulating Janus kinase 2/Signal transducer and activator of transcription 5A (JAK2/STAT5) signaling and SREBP1 expression [171]. Thus, VAD and hyperinsulinemia may synergize to activate HSC and promote fibrosis in NAFLD.

## 8. Vitamin A Therapy in NAFLD

Despite extensive historical and recent evidence that (1) vitamin A metabolism is disturbed in obesity and NAFLD and (2) vitamin A metabolites, especially atRA and synthetic RAR ligands, have beneficial effects on hepatic lipid metabolism and obesity-induced NAFLD in animal models, no clinical trials are ongoing to evaluate their therapeutic potential in patients. Instead, multiple trials are being performed to test the therapeutic value of synthetic ligands that modulate the activity of other nuclear receptors that control hepatic glucose and lipid metabolism, such as PPARα, PPARβ/δ, PPARγ, and FXR, all dimerization partners of RXRα. Also, variants of RAR-controlled FGF19 and FGF21 are in phase II clinical trials for the treatment of NAFLD [172]. As outlined above, vitamin A metabolism is heavily disturbed in NAFLD and will affect the activation status of RXRs and RARs. As an additional result, it will also modulate the activity of PPARs and FXR via the heterodimer partner RXR. Thus, the synthetic ligands for these receptors that are currently in clinical trials for the treatment of NAFLD and/or NASH may only bear partial effects because of impaired activation of the heterodimer partner RXR. Re-establishing proper levels of vitamin A metabolites, either systemically or directed to the liver, may have therapeutic value on its own, or potentiate the therapeutic effect of ligands of PPARs and/or FXR. Moreover, vitamin A metabolites also regulate bile acid synthesis directly via RXR- and RAR-mediated regulation of SHP and FGF15/19, as well as indirectly via RXR/FXR [155,173,174,175,176], which may feedback into FXR-mediated regulation of lipid and glucose metabolism.

In addition, retinaldehyde was identified about 10 years ago as promising compound to treat diet-induced obesity and diabetes in animal models. *Raldh1−/− mice*, which accumulate retinaldehyde in tissues, showed reduced hepatic lipid accumulation compared to wild-type (WT) mice when fed a high-fat diet (HFD) [161]. However, no application of retinaldehyde or RALDH inhibitors in NAFLD patients has been reported so far or is in the early phase of clinical testing.

Fenretinide (4-hydroxy(phenyl)retinamide; 4-HPR) is a synthetic retinoid that has been extensively studied for its potential therapeutic action against cancers, especially breast cancer, non-small cell lung cancer, neuroblastoma, and prostate cancer. Both RXR- and RAR-dependent and independent mechanisms have been proposed to underlie the therapeutic action of fenretinide, including inhibition of cell proliferation and the induction of apoptosis in cancer cells [177,178,179]. Fenretinide is well tolerated, with limited side effects in daily treatment regimens for five years or more [180,181]. Fenretinide also improves symptoms of diet-induced obesity, insulin resistance, and NAFLD. In addition, fenretinide improved insulin sensitivity and decreased serum leptin levels in a clinical trial in overweight women [182]. Fenretinide reduces circulating RBP4 levels, but the key importance of this effect in the therapeutic action of fenretinide is controversial, as it also prevents and/or reverses obesity, insulin resistance, and hepatic steatosis in *Rbp4* knockout mice on HFD [183]. Indeed, additional RAR-dependent and -independent mechanisms have also been identified that may contribute to the therapeutic effect of fenretinide in obesity-related pathologies, including enhanced mitochondrial and peroxisomal β-oxidation [184,185,186,187,188], ER-stress-mediated degradation of SCD1 [189], inhibition of ceramide synthesis, enhanced reactive oxygen species (ROS) production [184,190], enhanced retinoid signaling [191], and inhibition of hepatic FGF21 expression [192]. The many clinical trials aimed at evaluating fenretinide therapy in cancer have, however, shown that it dose-dependently reduces plasma retinol levels up to 90% compared to baseline pre-therapy, leading to vitamin A deficiency and impaired dark-adaptation as a regularly observed adverse effect [193,194,195,196,197,198,199,200,201,202,203,204,205,206,207,208,209,210,211,212]. The drop in circulating retinol cannot be prevented by vitamin A supplementation [196]. The retinol-lowering effects of fenretinide may be particular relevant for patients with metabolic syndrome and diabetes as plasma retinol levels are already low in those patents. A phase 2 clinical trial to evaluate the insulin-sensitizing effect of fenretinide in subjects with insulin resistance and NAFLD was initiated in 2007, but no results have been reported yet [213].

In summary, both natural and synthetic retinoids show important potential for the treatment of NAFLD and associated syndromes, like diabetes. However, these compounds act also on many other biological processes. Thus, tissue-specific targeting and/or characterization of derivatives that selectively modulate specific pathways may be required to arrive at a safe and effective treatment for NAFLD.

## 9. Conclusions

It is evident that hepatic glucose and lipid metabolism are regulated by vitamin A metabolites at many different levels. Moreover, disease progression within the NAFLD spectrum to NASH, cirrhosis, and cancer is associated with declining circulating and hepatic retinol levels. This is not necessarily true for hepatic retinyl esters as individuals homozygous for the PNPLA3-I148M risk allele are predisposed to NAFLD and disease progression, while their hepatic retinyl palmitate levels are increased compared to NAFLD-I148 (protective allele) carriers. It is unknown whether this shift from retinol to retinyl esters is more common in NALFD patients. Thus, we still lack important knowledge on hepatic vitamin A metabolism and the true meaning of VAD in NAFLD. To specify a few open questions: (1) are retinyl ester stores depleted in NAFLD or is retinol release from such stores impaired? (2) How can PNPLA3-I148M predispose for fibrosis while it leads to increased hepatic retinyl ester levels? (3) What is the contribution of hepatocytes and stellate cells to impaired retinol metabolism in NAFLD? (4) What is the absolute contribution of adipose-derived lipids and de novo lipogenesis in NAFLD and how is this controlled by vitamin A metabolites? (5) Why do circulating retinol levels stay low or even further decline after bariatric surgery, even under impressive weight loss and/or vitamin A supplementation therapy? (6) Is the therapeutic efficacy of nuclear receptor ligands that are currently under investigation for NAFLD limited by impaired vitamin A metabolism in the liver? (7) Does VAD actually contribute to the development of fatty liver?

With respect to the latter: Severe VAD in lean rats decreases serum triacylglycerol, cholesterol, and HDL cholesterol levels as well as hepatic phospholipids compared to VA-sufficient animals [214]. Expression of acetyl-CoA carboxylase decreased, suggesting impaired fatty acid synthesis, while mitochondrial fatty acid β-oxidation was enhanced. However, (free) cholesterol levels were enhanced in the hearts of VAD rats [215], as well as concentrations of triglycerides, total cholesterol, free and esterified cholesterol, and phospholipids in the aorta [216]. In contrast, VAD in mice appears to have the opposite effect. Hepatic TG levels are enhanced in VAD mice compared to control animals, which is associated with strongly reduced expression of PPARα and genes involved in mitochondrial and peroxisomal β-oxidation [217]. Thus, it also remains unclear whether VAD contributes to hepatic steatosis, and human data are so far lacking on this topic.

Thus, there is still a lot to learn about vitamin A metabolism in the liver in healthy and pathological conditions, which hopefully will reveal novel therapeutic targets for the treatment of NAFLD, in particular to prevent pathological conditions caused by NASH, cirrhosis, and hepatocellular carcinoma.

## References

[1] Blomhoff R., Blomhoff H.K. (2006). Overview of retinoid metabolism and function. J. Neurobiol..

[2] Lo C.S., Wahlqvist M.L., Horie Y. (1996). Determination of retinoic acid and retinol at physiological concentration by HPLC in Caucasians and Japanese women. Asia Pac. J. Clin. Nutr..

[3] Weber D., Grune T. (2012). The contribution of β-carotene to vitamin A supply of humans. Mol. Nutr. Food Res..

[4] Bar-El Dadon S., Reifen R. (2017). Vitamin A and the epigenome. Crit. Rev. Food Sci. Nutr..

[5] Zhong M., Kawaguchi R., Kassai M., Sun H. (2012). Retina, retinol, retinal and the natural history of vitamin A as a light sensor. Nutrients.

[6] Comptour A., Rouzaire M., Belville C., Bouvier D., Gallot D., Blanchon L., Sapin V. (2016). Nuclear retinoid receptors and pregnancy: Placental transfer, functions, and pharmacological aspects. Cell. Mol. Life Sci..

[7] Tanumihardjo S.A., Russell R.M., Stephensen C.B., Gannon B.M., Craft N.E., Haskell M.J., Lietz G., Schulze K., Raiten D.J. (2016). Biomarkers of Nutrition for Development (BOND)-Vitamin A Review. J. Nutr..

[8] Cunningham T.J., Duester G. (2015). Mechanisms of retinoic acid signalling and its roles in organ and limb development. Nat. Rev. Mol. Cell Biol..

[9] Bono M.R., Tejon G., Flores-Santibañez F., Fernandez D., Rosemblatt M., Sauma D. (2016). Retinoic Acid as a Modulator of T Cell Immunity. Nutrients.

[10] Huang P., Chandra V., Rastinejad F. (2014). Retinoic acid actions through mammalian nuclear receptors. Chem. Rev..

[11] Saeed A., Hoekstra M., Hoeke M.O., Heegsma J., Faber K.N. (2017). The interrelationship between bile acid and vitamin A homeostasis. Biochim. Biophys. Acta.

[12] Zhang R., Wang Y., Li R., Chen G. (2015). Transcriptional Factors Mediating Retinoic Acid Signals in the Control of Energy Metabolism. Int. J. Mol. Sci..

[13] Azaïs-Braesco V., Pascal G. (2000). Vitamin A in pregnancy: Requirements and safety limits. Am. J. Clin. Nutr..

[14] Andrews W.S., Pau C.M., Chase H.P., Foley L.C., Lilly J.R. (1981). Fat soluble vitamin deficiency in biliary atresia. J. Pediatr. Surg..

[15] Mourey M.S., Siegenthaler G., Amédée-Manesme O. (1990). Regulation of metabolism of retinol-binding protein by vitamin A status in children with biliary atresia. Am. J. Clin. Nutr..

[16] Herlong H.F., Russell R.M., Maddrey W.C. (1981). Vitamin A and zinc therapy in primary biliary cirrhosis. Hepatology.

[17] Walt R.P., Kemp C.M., Lyness L., Bird A.C., Sherlock S. (1984). Vitamin A treatment for night blindness in primary biliary cirrhosis. Br. Med. J..

[18] Phillips J.R., Angulo P., Petterson T., Lindor K.D. (2001). Fat-soluble vitamin levels in patients with primary biliary cirrhosis. Am. J. Gastroenterol..

[19] Jorgensen R.A., Lindor K.D., Sartin J.S., LaRusso N.F., Wiesner R.H. (1995). Serum lipid and fat-soluble vitamin levels in primary sclerosing cholangitis. J. Clin. Gastroenterol..

[20] Bitetto D., Bortolotti N., Falleti E., Vescovo S., Fabris C., Fattovich G., Cussigh A., Cmet S., Fornasiere E., Ceriani E. (2013). Vitamin A deficiency is associated with hepatitis C virus chronic infection and with unresponsiveness to interferon-based antiviral therapy. Hepatology.

[21] Majumdar S.K., Shaw G.K., Thomson A.D. (1983). Vitamin A utilization status in chronic alcoholic patients. Int. J. Vitam. Nutr. Res..

[22] Ray M.B., Mendenhall C.L., French S.W., Gartside P.S. (1988). Serum vitamin A deficiency and increased intrahepatic expression of cytokeratin antigen in alcoholic liver disease. Hepatology.

[23] Suano de Souza F.I., Silverio Amancio O.M., Saccardo Sarni R.O., Sacchi Pitta T., Fernandes A.P., Affonso Fonseca F.L., Hix S., Ramalho R.A. (2008). Non-alcoholic fatty liver disease in overweight children and its relationship with retinol serum levels. Int. J. Vitam. Nutr. Res..

[24] Botella-Carretero J.I., Balsa J.A., Vázquez C., Peromingo R., Díaz-Enriquez M., Escobar-Morreale H.F. (2010). Retinol and alpha-tocopherol in morbid obesity and nonalcoholic fatty liver disease. Obes. Surg..

[25] Chaves G.V., Pereira S.E., Saboya C.J., Spitz D., Rodrigues C.S., Ramalho A. (2014). Association between liver vitamin A reserves and severity of nonalcoholic fatty liver disease in the class III obese following bariatric surgery. Obes. Surg..

[26] Van den Berg E.H., Amini M., Schreuder T.C., Dullaart R.P., Faber K.N., Alizadeh B.Z., Blokzijl H. (2017). Prevalence and determinants of non-alcoholic fatty liver disease in lifelines: A large Dutch population cohort. PLoS ONE.

[27] Hannah W.N., Harrison S.A. (2016). Lifestyle and Dietary Interventions in the Management of Nonalcoholic Fatty Liver Disease. Dig. Dis. Sci..

[28] Nguyen V., George J. (2015). Nonalcoholic Fatty Liver Disease Management: Dietary and Lifestyle Modifications. Semin. Liver Dis..

[29] World Health Organization (1996). Indicators for Assessing Vitamin A Deficiency and Their Application in Monitoring and Evaluating Intervention Programmes.

[30] De Souza Valente da Silva L., Valeria da Veiga G., Ramalho R.A. (2007). Association of serum concentrations of retinol and carotenoids with overweight in children and adolescents. Nutrition.

[31] Neuhouser M.L., Rock C.L., Eldridge A.L., Kristal A.R., Patterson R.E., Cooper D.A., Neumark-Sztainer D., Cheskin L.J., Thornquist M.D. (2001). Serum concentrations of retinol, alpha-tocopherol and the carotenoids are influenced by diet, race and obesity in a sample of healthy adolescents. J. Nutr..

[32] Malik N.A. (2016). Solubilization and Interaction Studies of Bile Salts with Surfactants and Drugs: A Review. Appl. Biochem. Biotechnol..

[33] Nordskog B.K., Phan C.T., Nutting D.F., Tso P. (2001). An examination of the factors affecting intestinal lymphatic transport of dietary lipids. Adv. Drug Deliv. Rev..

[34] During A., Harrison E.H. (2007). Mechanisms of provitamin A (carotenoid) and vitamin A (retinol) transport into and out of intestinal Caco-2 cells. J. Lipid Res..

[35] Hussain M.M. (2000). A proposed model for the assembly of chylomicrons. Atherosclerosis.

[36] Ishibashi S., Perrey S., Chen Z., Osuga J., Shimada M., Ohashi K., Harada K., Yazaki Y., Yamada N. (1996). Role of the low density lipoprotein (LDL) receptor pathway in the metabolism of chylomicron remnants. A quantitative study in knockout mice lacking the LDL receptor, apolipoprotein E, or both. J. Biol. Chem..

[37] Linke T., Dawson H., Harrison E.H. (2005). Isolation and characterization of a microsomal acid retinyl ester hydrolase. J. Biol. Chem..

[38] Ronne H., Ocklind C., Wiman K., Rask L., Obrink B., Peterson P.A. (1983). Ligand-dependent regulation of intracellular protein transport: Effect of vitamin A on the secretion of the retinol-binding protein. J. Cell Biol..

[39] Dixon J.L., Goodman D.S. (1987). Studies on the metabolism of retinol-binding protein by primary hepatocytes from retinol-deficient rats. J. Cell. Physiol..

[40] Bellovino D., Lanyau Y., Garaguso I., Amicone L., Cavallari C., Tripodi M., Gaetani S. (1999). MMH cells: An in vitro model for the study of retinol-binding protein secretion regulated by retinol. J. Cell. Physiol..

[41] Sun H., Kawaguchi R. (2011). The membrane receptor for plasma retinol-binding protein, a new type of cell-surface receptor. Int. Rev. Cell Mol. Biol..

[42] Senoo H. (2004). Structure and function of hepatic stellate cells. Med. Electron Microsc..

[43] Wongsiriroj N., Jiang H., Piantedosi R., Yang K.J.Z., Kluwe J., Schwabe R.F., Ginsberg H., Goldberg I.J., Blaner W.S. (2014). Genetic dissection of retinoid esterification and accumulation in the liver and adipose tissue. J. Lipid Res..

[44] Taschler U., Schreiber R., Chitraju C., Grabner G.F., Romauch M., Wolinski H., Haemmerle G., Breinbauer R., Zechner R., Lass A. (2015). Adipose triglyceride lipase is involved in the mobilization of triglyceride and retinoid stores of hepatic stellate cells. Biochim. Biophys. Acta.

[45] Pirazzi C., Valenti L., Motta B.M., Pingitore P., Hedfalk K., Mancina R.M., Burza M.A., Indiveri C., Ferro Y., Montalcini T. (2014). PNPLA3 has retinyl-palmitate lipase activity in human hepatic stellate cells. Hum. Mol. Genet..

[46] Mondul A., Mancina R.M., Merlo A., Dongiovanni P., Rametta R., Montalcini T., Valenti L., Albanes D., Romeo S. (2015). PNPLA3 I148M Variant Influences Circulating Retinol in Adults with Nonalcoholic Fatty Liver Disease or Obesity. J. Nutr..

[47] Mello T., Nakatsuka A., Fears S., Davis W., Tsukamoto H., Bosron W.F., Sanghani S.P. (2008). Expression of carboxylesterase and lipase genes in rat liver cell-types. Biochem. Biophys. Res. Commun..

[48] Pang W., Zhang Y., Wang S., Jia A., Dong W., Cai C., Hua Z., Zhang J. (2011). The *mPlrp2* and *mClps* genes are involved in the hydrolysis of retinyl esters in the mouse liver. J. Lipid Res..

[49] Kim H., Lee K.-W., Lee K., Seo S., Park M.-Y., Ahn S.W., Hong S.K., Yoon K.C., Kim H.-S., Choi Y. (2017). Effect of PNPLA3 I148M polymorphism on histologically proven non-alcoholic fatty liver disease in liver transplant recipients. Hepatol. Res..

[50] Krawczyk M., Jiménez-Agüero R., Alustiza J.M., Emparanza J.I., Perugorria M.J., Bujanda L., Lammert F., Banales J.M. (2016). PNPLA3 p.I148M variant is associated with greater reduction of liver fat content after bariatric surgery. Surg. Obes. Relat. Dis..

[51] Xia M.-F., Ling Y., Bian H., Lin H.-D., Yan H.-M., Chang X.-X., Li X.-M., Ma H., Wang D., Zhang L.-S. (2016). I148M variant of PNPLA3 increases the susceptibility to non-alcoholic fatty liver disease caused by obesity and metabolic disorders. Aliment. Pharmacol. Ther..

[52] Bell H., Nilsson A., Norum K.R., Pedersen L.B., Raknerud N., Rasmussen M. (1989). Retinol and retinyl esters in patients with alcoholic liver disease. J. Hepatol..

[53] Villaça Chaves G., Pereira S.E., Saboya C.J., Ramalho A. (2008). Non-alcoholic fatty liver disease and its relationship with the nutritional status of vitamin A in individuals with class III obesity. Obes. Surg..

[54] Beydoun M.A., Shroff M.R., Chen X., Beydoun H.A., Wang Y., Zonderman A.B. (2011). Serum antioxidant status is associated with metabolic syndrome among U.S. adults in recent national surveys. J. Nutr..

[55] Pereira S.E., Saboya C.J., Saunders C., Ramalho A. (2012). Serum levels and liver store of retinol and their association with night blindness in individuals with class III obesity. Obes. Surg..

[56] Beydoun M.A., Canas J.A., Beydoun H.A., Chen X., Shroff M.R., Zonderman A.B. (2012). Serum antioxidant concentrations and metabolic syndrome are associated among U.S. adolescents in recent national surveys. J. Nutr..

[57] Lefebvre P., Letois F., Sultan A., Nocca D., Mura T., Galtier F. (2014). Nutrient deficiencies in patients with obesity considering bariatric surgery: A cross-sectional study. Surg. Obes. Relat. Dis..

[58] Teske M., Melges A.P., de Souza F.I., Fonseca F.L., Sarni R.O. (2014). Plasma concentrations of retinol in obese children and adolescents: Relationship to metabolic syndrome components. Rev. Paul. Pediatr..

[59] Wolf E., Utech M., Stehle P., Büsing M., Stoffel-Wagner B., Ellinger S. (2015). Preoperative micronutrient status in morbidly obese patients before undergoing bariatric surgery: Results of a cross-sectional study. Surg. Obes. Relat. Dis..

[60] Liu Y., Chen H., Mu D., Fan J., Song J., Zhong Y., Li D., Xia M. (2016). Circulating Retinoic Acid Levels and the Development of Metabolic Syndrome. J. Clin. Endocrinol. Metab..

[61] Wei X., Peng R., Cao J., Kang Y., Qu P., Liu Y., Xiao X., Li T. (2016). Serum vitamin A status is associated with obesity and the metabolic syndrome among school-age children in Chongqing, China. Asia Pac. J. Clin. Nutr..

[62] Godala M.M., Materek-Kuśmierkiewicz I., Moczulski D., Rutkowski M., Szatko F., Gaszyńska E., Tokarski S., Kowalski J. (2017). The risk of plasma vitamin A, C, E and D deficiency in patients with metabolic syndrome: A case-control study. Adv. Clin. Exp. Med..

[63] Trasino S.E., Tang X.-H., Jessurun J., Gudas L.J. (2015). Obesity Leads to Tissue, but not Serum Vitamin A Deficiency. Sci. Rep..

[64] Liu Y., Chen H., Wang J., Zhou W., Sun R., Xia M. (2015). Association of serum retinoic acid with hepatic steatosis and liver injury in nonalcoholic fatty liver disease. Am. J. Clin. Nutr..

[65] Ashla A.A., Hoshikawa Y., Tsuchiya H., Hashiguchi K., Enjoji M., Nakamuta M., Taketomi A., Maehara Y., Shomori K., Kurimasa A. (2010). Genetic analysis of expression profile involved in retinoid metabolism in non-alcoholic fatty liver disease. Hepatol. Res..

[66] Clemente C., Elba S., Buongiorno G., Berloco P., Guerra V., Di Leo A. (2002). Serum retinol and risk of hepatocellular carcinoma in patients with child-Pugh class A cirrhosis. Cancer Lett..

[67] Newsome P.N., Beldon I., Moussa Y., Delahooke T.E., Poulopoulos G., Hayes P.C., Plevris J.N. (2000). Low serum retinol levels are associated with hepatocellular carcinoma in patients with chronic liver disease. Aliment. Pharmacol. Ther..

[68] Yanagitani A., Yamada S., Yasui S., Shimomura T., Murai R., Murawaki Y., Hashiguchi K., Kanbe T., Saeki T., Ichiba M. (2004). Retinoic acid receptor alpha dominant negative form causes steatohepatitis and liver tumors in transgenic mice. Hepatology.

[69] Yang Q., Graham T.E., Mody N., Preitner F., Peroni O.D., Zabolotny J.M., Kotani K., Quadro L., Kahn B.B. (2005). Serum retinol binding protein 4 contributes to insulin resistance in obesity and type 2 diabetes. Nature.

[70] Haider D.G., Schindler K., Prager G., Bohdjalian A., Luger A., Wolzt M., Ludvik B. (2007). Serum retinol-binding protein 4 is reduced after weight loss in morbidly obese subjects. J. Clin. Endocrinol. Metab..

[71] Reinehr T., Stoffel-Wagner B., Roth C.L. (2008). Retinol-binding protein 4 and its relation to insulin resistance in obese children before and after weight loss. J. Clin. Endocrinol. Metab..

[72] Tajtáková M., Semanová Z., Ivancová G., Petrovicová J., Donicová V., Zemberová E. (2007). Serum level of retinol-binding protein 4 in obese patients with insulin resistance and in patients with type 2 diabetes treated with metformin. Vnitr. Lek..

[73] Ulgen F., Herder C., Kühn M.C., Willenberg H.S., Schott M., Scherbaum W.A., Schinner S. (2010). Association of serum levels of retinol-binding protein 4 with male sex but not with insulin resistance in obese patients. Arch. Physiol. Biochem..

[74] Broch M., Vendrell J., Ricart W., Richart C., Fernández-Real J.-M. (2007). Circulating retinol-binding protein-4, insulin sensitivity, insulin secretion, and insulin disposition index in obese and nonobese subjects. Diabetes Care.

[75] Gómez-Ambrosi J., Rodríguez A., Catalán V., Ramírez B., Silva C., Rotellar F., Gil M.J., Salvador J., Frühbeck G. (2008). Serum retinol-binding protein 4 is not increased in obesity or obesity-associated type 2 diabetes mellitus, but is reduced after relevant reductions in body fat following gastric bypass. Clin. Endocrinol..

[76] Kanaka-Gantenbein C., Margeli A., Pervanidou P., Sakka S., Mastorakos G., Chrousos G.P., Papassotiriou I. (2008). Retinol-binding protein 4 and lipocalin-2 in childhood and adolescent obesity: When children are not just “small adults”. Clin. Chem..

[77] Alkhouri N., Lopez R., Berk M., Feldstein A.E. (2009). Serum retinol-binding protein 4 levels in patients with nonalcoholic fatty liver disease. J. Clin. Gastroenterol..

[78] Comerford K.B., Buchan W., Karakas S.E. (2014). The effects of weight loss on FABP4 and RBP4 in obese women with metabolic syndrome. Horm. Metab. Res..

[79] Aeberli I., Biebinger R., Lehmann R., L’allemand D., Spinas G.A., Zimmermann M.B. (2007). Serum retinol-binding protein 4 concentration and its ratio to serum retinol are associated with obesity and metabolic syndrome components in children. J. Clin. Endocrinol. Metab..

[80] Mills J.P., Furr H.C., Tanumihardjo S.A. (2008). Retinol to retinol-binding protein (RBP) is low in obese adults due to elevated apo-RBP. Exp. Biol. Med..

[81] Erikstrup C., Mortensen O.H., Nielsen A.R., Fischer C.P., Plomgaard P., Petersen A.M., Krogh-Madsen R., Lindegaard B., Erhardt J.G., Ullum H. (2009). RBP-to-retinol ratio, but not total RBP, is elevated in patients with type 2 diabetes. Diabetes Obes. Metab..

[82] Henze A., Frey S.K., Raila J., Tepel M., Scholze A., Pfeiffer A.F.H., Weickert M.O., Spranger J., Schweigert F.J. (2008). Evidence that kidney function but not type 2 diabetes determines retinol-binding protein 4 serum levels. Diabetes.

[83] Zwolak A., Szuster-Ciesielska A., Daniluk J., Semeniuk J., Kandefer-Szerszen M. (2016). Chemerin, retinol binding protein-4, cytokeratin-18 and transgelin-2 presence in sera of patients with non-alcoholic liver fatty disease. Ann. Hepatol..

[84] Rahimlou M., Mirzaei K., Keshavarz S.A., Hossein-Nezhad A. (2016). Association of circulating adipokines with metabolic dyslipidemia in obese versus non-obese individuals. Diabetes Metab. Syndr..

[85] Chu C.-H., Lam H.-C., Lee J.-K., Lu C.-C., Sun C.-C., Cheng H.-J., Wang M.-C., Chuang M.-J. (2011). Elevated serum retinol-binding protein 4 concentrations are associated with chronic kidney disease but not with the higher carotid intima-media thickness in type 2 diabetic subjects. Endocr. J..

[86] Wu H., Jia W., Bao Y., Lu J., Zhu J., Wang R., Chen Y., Xiang K. (2008). Serum retinol binding protein 4 and nonalcoholic fatty liver disease in patients with type 2 diabetes mellitus. Diabetes Res. Clin. Pract..

[87] Thompson S.J., Sargsyan A., Lee S.-A., Yuen J.J., Cai J., Smalling R., Ghyselinck N., Mark M., Blaner W.S., Graham T.E. (2017). Hepatocytes Are the Principal Source of Circulating RBP4 in Mice. Diabetes.

[88] Lee S.-A., Yuen J.J., Jiang H., Kahn B.B., Blaner W.S. (2016). Adipocyte-specific overexpression of retinol-binding protein 4 causes hepatic steatosis in mice. Hepatology.

[89] Schina M., Koskinas J., Tiniakos D., Hadziyannis E., Savvas S., Karamanos B., Manesis E., Archimandritis A. (2009). Circulating and liver tissue levels of retinol-binding protein-4 in non-alcoholic fatty liver disease. Hepatol. Res..

[90] De Luis D.A., Pacheco D., Izaola O., Terroba M.C., Cuellar L., Martin T. (2008). Clinical results and nutritional consequences of biliopancreatic diversion: Three years of follow-up. Ann. Nutr. Metab..

[91] Aasheim E.T., Björkman S., Søvik T.T., Engström M., Hanvold S.E., Mala T., Olbers T., Bøhmer T. (2009). Vitamin status after bariatric surgery: A randomized study of gastric bypass and duodenal switch. Am. J. Clin. Nutr..

[92] Pereira S., Saboya C., Chaves G., Ramalho A. (2009). Class III obesity and its relationship with the nutritional status of vitamin A in pre- and postoperative gastric bypass. Obes. Surg..

[93] Cuesta M., Pelaz L., Pérez C., Torrejón M.J., Cabrerizo L., Matía P., Pérez-Ferre N., Sánchez-Pernaute A., Torres A., Rubio M.A. (2014). Fat-soluble vitamin deficiencies after bariatric surgery could be misleading if they are not appropriately adjusted. Nutr. Hosp..

[94] Silva J.S., Chaves G.V., Stenzel A.P., Pereira S.E., Saboya C.J., Ramalho A. (2017). Improvement of anthropometric and biochemical, but not of vitamin A, status in adolescents who undergo Roux-en-Y gastric bypass: A 1-year follow up study. Surg. Obes. Relat. Dis..

[95] Søvik T.T., Aasheim E.T., Taha O., Engström M., Fagerland M.W., Björkman S., Kristinsson J., Birkeland K.I., Mala T., Olbers T. (2011). Weight loss, cardiovascular risk factors, and quality of life after gastric bypass and duodenal switch: A randomized trial. Ann. Intern. Med..

[96] Slater G.H., Ren C.J., Siegel N., Williams T., Barr D., Wolfe B., Dolan K., Fielding G.A. (2004). Serum fat-soluble vitamin deficiency and abnormal calcium metabolism after malabsorptive bariatric surgery. J. Gastrointest. Surg..

[97] Nett P., Borbély Y., Kröll D. (2016). Micronutrient Supplementation after Biliopancreatic Diversion with Duodenal Switch in the Long Term. Obes. Surg..

[98] Bolckmans R., Himpens J. (2016). Long-term (>10 Years) Outcome of the Laparoscopic Biliopancreatic Diversion with Duodenal Switch. Ann. Surg..

[99] Gilchrist H., Taranath D.A., Gole G.A. (2010). Ocular malformation in a newborn secondary to maternal hypovitaminosis A. J. AAPOS.

[100] Gascoin G., Gerard M., Sallé A., Becouarn G., Rouleau S., Sentilhes L., Coutant R. (2017). Risk of low birth weight and micronutrient deficiencies in neonates from mothers after gastric bypass: A case control study. Surg. Obes. Relat. Dis..

[101] Pereira S., Saboya C., Ramalho A. (2013). Impact of different protocols of nutritional supplements on the status of vitamin A in class III obese patients after Roux-en-Y gastric bypass. Obes. Surg..

[102] Topart P., Becouarn G., Sallé A., Ritz P. (2014). Biliopancreatic diversion requires multiple vitamin and micronutrient adjustments within 2 years of surgery. Surg. Obes. Relat. Dis..

[103] Fok J.S., Li J.Y., Yong T.Y. (2012). Visual deterioration caused by vitamin A deficiency in patients after bariatric surgery. Eat. Weight Disord..

[104] Vitkova M., Klimcakova E., Kovacikova M., Valle C., Moro C., Polak J., Hanacek J., Capel F., Viguerie N., Richterova B. (2007). Plasma levels and adipose tissue messenger ribonucleic acid expression of retinol-binding protein 4 are reduced during calorie restriction in obese subjects but are not related to diet-induced changes in insulin sensitivity. J. Clin. Endocrinol. Metab..

[105] Mitterberger M.C., Mattesich M., Klaver E., Lechner S., Engelhardt T., Larcher L., Pierer G., Piza-Katzer H., Zwerschke W. (2010). Adipokine profile and insulin sensitivity in formerly obese women subjected to bariatric surgery or diet-induced long-term caloric restriction. J. Gerontol. A Biol. Sci. Med. Sci..

[106] Klempel M.C., Varady K.A. (2011). Reliability of leptin, but not adiponectin, as a biomarker for diet-induced weight loss in humans. Nutr. Rev..

[107] Wang P., Zhang R.-Y., Song J., Guan Y.-F., Xu T.-Y., Du H., Viollet B., Miao C.-Y. (2012). Loss of AMP-activated protein kinase-α2 impairs the insulin-sensitizing effect of calorie restriction in skeletal muscle. Diabetes.

[108] Romeo S., Kozlitina J., Xing C., Pertsemlidis A., Cox D., Pennacchio L.A., Boerwinkle E., Cohen J.C., Hobbs H.H. (2008). Genetic variation in PNPLA3 confers susceptibility to nonalcoholic fatty liver disease. Nat. Genet..

[109] Valenti L., Al-Serri A., Daly A.K., Galmozzi E., Rametta R., Dongiovanni P., Nobili V., Mozzi E., Roviaro G., Vanni E. (2010). Homozygosity for the patatin-like phospholipase-3/adiponutrin I148M polymorphism influences liver fibrosis in patients with nonalcoholic fatty liver disease. Hepatology.

[110] Sookoian S., Pirola C.J. (2011). Meta-analysis of the influence of I148M variant of patatin-like phospholipase domain containing 3 gene (PNPLA3) on the susceptibility and histological severity of nonalcoholic fatty liver disease. Hepatology.

[111] Liu Y.-L., Patman G.L., Leathart J.B.S., Piguet A.-C., Burt A.D., Dufour J.-F., Day C.P., Daly A.K., Reeves H.L., Anstee Q.M. (2014). Carriage of the PNPLA3 rs738409 C >G polymorphism confers an increased risk of non-alcoholic fatty liver disease associated hepatocellular carcinoma. J. Hepatol..

[112] Severson T.J., Besur S., Bonkovsky H.L. (2016). Genetic factors that affect nonalcoholic fatty liver disease: A systematic clinical review. World J. Gastroenterol..

[113] Li J.Z., Huang Y., Karaman R., Ivanova P.T., Brown H.A., Roddy T., Castro-Perez J., Cohen J.C., Hobbs H.H. (2012). Chronic overexpression of PNPLA3I148M in mouse liver causes hepatic steatosis. J. Clin. Investig..

[114] Lallukka S., Yki-Järvinen H. (2016). Non-alcoholic fatty liver disease and risk of type 2 diabetes. Best Pract. Res. Clin. Endocrinol. Metab..

[115] Petäjä E.M., Yki-Järvinen H. (2016). Definitions of Normal Liver Fat and the Association of Insulin Sensitivity with Acquired and Genetic NAFLD—A Systematic Review. Int. J. Mol. Sci..

[116] Kovarova M., Königsrainer I., Königsrainer A., Machicao F., Häring H.-U., Schleicher E., Peter A. (2015). The Genetic Variant I148M in PNPLA3 Is Associated With Increased Hepatic Retinyl-Palmitate Storage in Humans. J. Clin. Endocrinol. Metab..

[117] Bruschi F.V., Claudel T., Tardelli M., Caligiuri A., Stulnig T.M., Marra F., Trauner M. (2017). The PNPLA3 I148M variant modulates the fibrogenic phenotype of human hepatic stellate cells. Hepatology.

[118] Papazyan R., Sun Z., Kim Y.H., Titchenell P.M., Hill D.A., Lu W., Damle M., Wan M., Zhang Y., Briggs E.R. (2016). Physiological Suppression of Lipotoxic Liver Damage by Complementary Actions of HDAC3 and SCAP/SREBP. Cell Metab..

[119] Softic S., Cohen D.E., Kahn C.R. (2016). Role of Dietary Fructose and Hepatic de novo Lipogenesis in Fatty Liver Disease. Dig. Dis. Sci..

[120] Iizuka K., Horikawa Y. (2008). ChREBP: A glucose-activated transcription factor involved in the development of metabolic syndrome. Endocr. J..

[121] Lalloyer F., Pedersen T.A., Gross B., Lestavel S., Yous S., Vallez E., Gustafsson J.-A., Mandrup S., Fiévet C., Staels B. (2009). Rexinoid bexarotene modulates triglyceride but not cholesterol metabolism via gene-specific permissivity of the RXR/LXR heterodimer in the liver. Arterioscler. Thromb. Vasc. Biol..

[122] Joseph S.B., Laffitte B.A., Patel P.H., Watson M.A., Matsukuma K.E., Walczak R., Collins J.L., Osborne T.F., Tontonoz P. (2002). Direct and indirect mechanisms for regulation of fatty acid synthase gene expression by liver X receptors. J. Biol. Chem..

[123] Kim S.C., Kim C.-K., Axe D., Cook A., Lee M., Li T., Smallwood N., Chiang J.Y.L., Hardwick J.P., Moore D.D. (2014). All-trans-retinoic acid ameliorates hepatic steatosis in mice by a novel transcriptional cascade. Hepatology.

[124] Watanabe M., Houten S.M., Wang L., Moschetta A., Mangelsdorf D.J., Heyman R.A., Moore D.D., Auwerx J. (2004). Bile acids lower triglyceride levels via a pathway involving FXR, SHP and SREBP-1c. J. Clin. Investig..

[125] Wuttge D.M., Romert A., Eriksson U., Törmä H., Hansson G.K., Sirsjö A. (2001). Induction of CD36 by all-trans retinoic acid: Retinoic acid receptor signaling in the pathogenesis of atherosclerosis. FASEB J..

[126] Han S., Sidell N. (2002). Peroxisome-proliferator-activated-receptor gamma (PPARgamma) independent induction of CD36 in THP-1 monocytes by retinoic acid. Immunology.

[127] Ishimoto K., Tachibana K., Sumitomo M., Omote S., Hanano I., Yamasaki D., Watanabe Y., Tanaka T., Hamakubo T., Sakai J. (2006). Identification of human low-density lipoprotein receptor as a novel target gene regulated by liver X receptor alpha. FEBS Lett..

[128] Yao Z. (2012). Human apolipoprotein C-III—A new intrahepatic protein factor promoting assembly and secretion of very low density lipoproteins. Cardiovasc. Hematol. Disord. Drug Targets.

[129] Lee H.-Y., Birkenfeld A.L., Jornayvaz F.R., Jurczak M.J., Kanda S., Popov V., Frederick D.W., Zhang D., Guigni B., Bharadwaj K.G. (2011). Apolipoprotein CIII overexpressing mice are predisposed to diet-induced hepatic steatosis and hepatic insulin resistance. Hepatology.

[130] Zhang R.-N., Zheng R.-D., Mi Y.-Q., Zhou D., Shen F., Chen G.-Y., Zhu C.-Y., Pan Q., Fan J.-G. (2016). APOC3 rs2070666 Is Associated with the Hepatic Steatosis Independently of PNPLA3 rs738409 in Chinese Han Patients with Nonalcoholic Fatty Liver Diseases. Dig. Dis. Sci..

[131] Lee S.J., Mahankali M., Bitar A., Zou H., Chao E., Nguyen H., Gonzalez J., Caballero D., Hull M., Wang D. (2017). A Novel Role for RARα Agonists as Apolipoprotein CIII Inhibitors Identified from High Throughput Screening. Sci. Rep..

[132] Vu-Dac N., Gervois P., Torra I.P., Fruchart J.C., Kosykh V., Kooistra T., Princen H.M., Dallongeville J., Staels B. (1998). Retinoids increase human apo C-III expression at the transcriptional level via the retinoid X receptor. Contribution to the hypertriglyceridemic action of retinoids. J. Clin. Investig..

[133] Caron S., Verrijken A., Mertens I., Samanez C.H., Mautino G., Haas J.T., Duran-Sandoval D., Prawitt J., Francque S., Vallez E. (2011). Transcriptional activation of apolipoprotein CIII expression by glucose may contribute to diabetic dyslipidemia. Arterioscler. Thromb. Vasc. Biol..

[134] Ong K.T., Mashek M.T., Bu S.Y., Greenberg A.S., Mashek D.G. (2011). Adipose triglyceride lipase is a major hepatic lipase that regulates triacylglycerol turnover and fatty acid signaling and partitioning. Hepatology.

[135] Jha P., Claudel T., Baghdasaryan A., Mueller M., Halilbasic E., Das S.K., Lass A., Zimmermann R., Zechner R., Hoefler G. (2014). Role of adipose triglyceride lipase (PNPLA2) in protection from hepatic inflammation in mouse models of steatohepatitis and endotoxemia. Hepatology.

[136] Harada K., Shen W.-J., Patel S., Natu V., Wang J., Osuga J., Ishibashi S., Kraemer F.B. (2003). Resistance to high-fat diet-induced obesity and altered expression of adipose-specific genes in HSL-deficient mice. Am. J. Physiol. Endocrinol. Metab..

[137] Reid B.N., Ables G.P., Otlivanchik O.A., Schoiswohl G., Zechner R., Blaner W.S., Goldberg I.J., Schwabe R.F., Chua S.C., Huang L.-S. (2008). Hepatic overexpression of hormone-sensitive lipase and adipose triglyceride lipase promotes fatty acid oxidation, stimulates direct release of free fatty acids, and ameliorates steatosis. J. Biol. Chem..

[138] Rakhshandehroo M., Sanderson L.M., Matilainen M., Stienstra R., Carlberg C., de Groot P.J., Müller M., Kersten S. (2007). Comprehensive analysis of PPARalpha-dependent regulation of hepatic lipid metabolism by expression profiling. PPAR Res..

[139] Karahashi M., Hoshina M., Yamazaki T., Sakamoto T., Mitsumoto A., Kawashima Y., Kudo N. (2013). Fibrates reduce triacylglycerol content by upregulating adipose triglyceride lipase in the liver of rats. J. Pharmacol. Sci..

[140] Deng T., Shan S., Li P.-P., Shen Z.-F., Lu X.-P., Cheng J., Ning Z.-Q. (2006). Peroxisome proliferator-activated receptor-gamma transcriptionally up-regulates hormone-sensitive lipase via the involvement of specificity protein-1. Endocrinology.

[141] Stenson B.M., Rydén M., Venteclef N., Dahlman I., Pettersson A.M.L., Mairal A., Aström G., Blomqvist L., Wang V., Jocken J.W.E. (2011). Liver X receptor (LXR) regulates human adipocyte lipolysis. J. Biol. Chem..

[142] Dubuquoy C., Robichon C., Lasnier F., Langlois C., Dugail I., Foufelle F., Girard J., Burnol A.-F., Postic C., Moldes M. (2011). Distinct regulation of adiponutrin/PNPLA3 gene expression by the transcription factors ChREBP and SREBP1c in mouse and human hepatocytes. J. Hepatol..

[143] Perttilä J., Huaman-Samanez C., Caron S., Tanhuanpää K., Staels B., Yki-Järvinen H., Olkkonen V.M. (2012). PNPLA3 is regulated by glucose in human hepatocytes, and its I148M mutant slows down triglyceride hydrolysis. Am. J. Physiol. Endocrinol. Metab..

[144] Kersten S., Stienstra R. (2017). The role and regulation of the peroxisome proliferator activated receptor alpha in human liver. Biochimie.

[145] Pawlak M., Lefebvre P., Staels B. (2015). Molecular mechanism of PPARα action and its impact on lipid metabolism, inflammation and fibrosis in non-alcoholic fatty liver disease. J. Hepatol..

[146] Amengual J., Ribot J., Bonet M.L., Palou A. (2010). Retinoic acid treatment enhances lipid oxidation and inhibits lipid biosynthesis capacities in the liver of mice. Cell. Physiol. Biochem..

[147] Amengual J., Petrov P., Bonet M.L., Ribot J., Palou A. (2012). Induction of carnitine palmitoyl transferase 1 and fatty acid oxidation by retinoic acid in HepG2 cells. Int. J. Biochem. Cell Biol..

[148] Patton A., Khan F.H., Kohli R. (2017). Impact of Fibroblast Growth Factors 19 and 21 in Bariatric Metabolism. Dig. Dis..

[149] Li Y., Wong K., Walsh K., Gao B., Zang M. (2013). Retinoic acid receptor β stimulates hepatic induction of fibroblast growth factor 21 to promote fatty acid oxidation and control whole-body energy homeostasis in mice. J. Biol. Chem..

[150] Cyphert H.A., Ge X., Kohan A.B., Salati L.M., Zhang Y., Hillgartner F.B. (2012). Activation of the farnesoid X receptor induces hepatic expression and secretion of fibroblast growth factor 21. J. Biol. Chem..

[151] Holt J.A., Luo G., Billin A.N., Bisi J., McNeill Y.Y., Kozarsky K.F., Donahee M., Wang D.Y., Mansfield T.A., Kliewer S.A. (2003). Definition of a novel growth factor-dependent signal cascade for the suppression of bile acid biosynthesis. Genes Dev..

[152] Inagaki T., Choi M., Moschetta A., Peng L., Cummins C.L., McDonald J.G., Luo G., Jones S.A., Goodwin B., Richardson J.A. (2005). Fibroblast growth factor 15 functions as an enterohepatic signal to regulate bile acid homeostasis. Cell Metab..

[153] Alvarez-Sola G., Uriarte I., Latasa M.U., Urtasun R., Bárcena-Varela M., Elizalde M., Jiménez M., Rodriguez-Ortigosa C.M., Corrales F.J., Fernández-Barrena M.G. (2017). Fibroblast Growth Factor 15/19 in Hepatocarcinogenesis. Dig. Dis..

[154] Degirolamo C., Sabbà C., Moschetta A. (2016). Therapeutic potential of the endocrine fibroblast growth factors FGF19, FGF21 and FGF23. Nat. Rev. Drug Discov..

[155] Jahn D., Sutor D., Dorbath D., Weiß J., Götze O., Schmitt J., Hermanns H.M., Geier A. (2016). Farnesoid X receptor-dependent and -independent pathways mediate the transcriptional control of human fibroblast growth factor 19 by vitamin A. Biochim. Biophys. Acta.

[156] Noy N. (2013). The one-two punch. Adipocyte.

[157] Noy N. (2016). Vitamin A in regulation of insulin responsiveness: Mini review. Proc. Nutr. Soc..

[158] Muenzner M., Tuvia N., Deutschmann C., Witte N., Tolkachov A., Valai A., Henze A., Sander L.E., Raila J., Schupp M. (2013). Retinol-binding protein 4 and its membrane receptor STRA6 control adipogenesis by regulating cellular retinoid homeostasis and retinoic acid receptor α activity. Mol. Cell. Biol..

[159] Canan Koch S.S., Dardashti L.J., Cesario R.M., Croston G.E., Boehm M.F., Heyman R.A., Nadzan A.M. (1999). Synthesis of retinoid X receptor-specific ligands that are potent inducers of adipogenesis in 3T3-L1 cells. J. Med. Chem..

[160] Kiefer F.W., Vernochet C., O’Brien P., Spoerl S., Brown J.D., Nallamshetty S., Zeyda M., Stulnig T.M., Cohen D.E., Kahn C.R. (2012). Retinaldehyde dehydrogenase 1 regulates a thermogenic program in white adipose tissue. Nat. Med..

[161] Ziouzenkova O., Orasanu G., Sharlach M., Akiyama T.E., Berger J.P., Viereck J., Hamilton J.A., Tang G., Dolnikowski G.G., Vogel S. (2007). Retinaldehyde represses adipogenesis and diet-induced obesity. Nat. Med..

[162] Ito K., Hao L., Wray A.E., Ross A.C. (2013). Lipid emulsion administered intravenously or orally attenuates triglyceride accumulation and expression of inflammatory markers in the liver of nonobese mice fed parenteral nutrition formula. J. Nutr..

[163] Abd Eldaim M.A., Matsuoka S., Okamatsu-Ogura Y., Kamikawa A., Ahmed M.M., Terao A., Nakajima K.-I., Kimura K. (2017). Retinoic acid modulates lipid accumulation glucose concentration dependently through inverse regulation of SREBP-1 expression in 3T3L1 adipocytes. Genes Cells.

[164] Trasino S.E., Benoit Y.D., Gudas L.J. (2015). Vitamin A Deficiency Causes Hyperglycemia and Loss of Pancreatic β-Cell Mass. J. Biol. Chem..

[165] Brun P.-J., Grijalva A., Rausch R., Watson E., Yuen J.J., Das B.C., Shudo K., Kagechika H., Leibel R.L., Blaner W.S. (2015). Retinoic acid receptor signaling is required to maintain glucose-stimulated insulin secretion and β-cell mass. FASEB J..

[166] Brun P.-J., Wongsiriroj N., Blaner W.S. (2016). Retinoids in the pancreas. Hepatobiliary Surg. Nutr..

[167] Trasino S.E., Tang X.-H., Jessurun J., Gudas L.J. (2016). Retinoic acid receptor β2 agonists restore glycaemic control in diabetes and reduce steatosis. Diabetes Obes. Metab..

[168] Svegliati-Baroni G., Ridolfi F., Di Sario A., Casini A., Marucci L., Gaggiotti G., Orlandoni P., Macarri G., Perego L., Benedetti A. (1999). Insulin and insulin-like growth factor-1 stimulate proliferation and type I collagen accumulation by human hepatic stellate cells: Differential effects on signal transduction pathways. Hepatology.

[169] Adachi M., Osawa Y., Uchinami H., Kitamura T., Accili D., Brenner D.A. (2007). The forkhead transcription factor FoxO1 regulates proliferation and transdifferentiation of hepatic stellate cells. Gastroenterology.

[170] Zhang J., Li S., Li J., Han C., Wang Z., Li C., Wang X., Liu Z., Wen J., Zheng L. (2014). Expression and significance of fat mass and obesity associated gene and forkhead transcription factor O1 in non-alcoholic fatty liver disease. Chin. Med. J..

[171] Yoneda A., Sakai-Sawada K., Niitsu Y., Tamura Y. (2016). Vitamin A and insulin are required for the maintenance of hepatic stellate cell quiescence. Exp. Cell Res..

[172] Rotman Y., Sanyal A.J. (2017). Current and upcoming pharmacotherapy for non-alcoholic fatty liver disease. Gut.

[173] Schmidt D.R., Holmstrom S.R., Fon Tacer K., Bookout A.L., Kliewer S.A., Mangelsdorf D.J. (2010). Regulation of bile acid synthesis by fat-soluble vitamins A and D. J. Biol. Chem..

[174] Hoeke M.O., Heegsma J., Hoekstra M., Moshage H., Faber K.N. (2014). Human FXR regulates SHP expression through direct binding to an LRH-1 binding site, independent of an IR-1 and LRH-1. PLoS ONE.

[175] Yang F., He Y., Liu H.-X., Tsuei J., Jiang X., Yang L., Wang Z.-T., Wan Y.-J. (2014). All-trans retinoic acid regulates hepatic bile acid homeostasis. Biochem. Pharmacol..

[176] Mamoon A., Subauste A., Subauste M.C., Subauste J. (2014). Retinoic acid regulates several genes in bile acid and lipid metabolism via upregulation of small heterodimer partner in hepatocytes. Gene.

[177] Hail N., Kim H.J., Lotan R. (2006). Mechanisms of fenretinide-induced apoptosis. Apoptosis.

[178] Brtko J. (2007). Role of retinoids and their cognate nuclear receptors in breast cancer chemoprevention. Cent. Eur. J. Public Health.

[179] Macis D., Gandini S., Guerrieri-Gonzaga A., Johansson H., Magni P., Ruscica M., Lazzeroni M., Serrano D., Cazzaniga M., Mora S. (2012). Prognostic effect of circulating adiponectin in a randomized 2 × 2 trial of low-dose tamoxifen and fenretinide in premenopausal women at risk for breast cancer. J. Clin. Oncol..

[180] Formelli F., Clerici M., Campa T., Di Mauro M.G., Magni A., Mascotti G., Moglia D., De Palo G., Costa A., Veronesi U. (1993). Five-year administration of fenretinide: Pharmacokinetics and effects on plasma retinol concentrations. J. Clin. Oncol..

[181] Decensi A., Johansson H., Miceli R., Mariani L., Camerini T., Cavadini E., Di Mauro M.G., Barreca A., Gonzaga A.G., Diani S. (2001). Long-term effects of fenretinide, a retinoic acid derivative, on the insulin-like growth factor system in women with early breast cancer. Cancer Epidemiol. Biomark. Prev..

[182] Johansson H., Gandini S., Guerrieri-Gonzaga A., Iodice S., Ruscica M., Bonanni B., Gulisano M., Magni P., Formelli F., Decensi A. (2008). Effect of fenretinide and low-dose tamoxifen on insulin sensitivity in premenopausal women at high risk for breast cancer. Cancer Res..

[183] Preitner F., Mody N., Graham T.E., Peroni O.D., Kahn B.B. (2009). Long-term Fenretinide treatment prevents high-fat diet-induced obesity, insulin resistance, and hepatic steatosis. Am. J. Physiol. Endocrinol. Metab..

[184] Mcilroy G.D., Tammireddy S.R., Maskrey B.H., Grant L., Doherty M.K., Watson D.G., Delibegović M., Whitfield P.D., Mody N. (2016). Fenretinide mediated retinoic acid receptor signalling and inhibition of ceramide biosynthesis regulates adipogenesis, lipid accumulation, mitochondrial function and nutrient stress signalling in adipocytes and adipose tissue. Biochem. Pharmacol..

[185] Rahmaniyan M., Curley R.W., Obeid L.M., Hannun Y.A., Kraveka J.M. (2011). Identification of dihydroceramide desaturase as a direct in vitro target for fenretinide. J. Biol. Chem..

[186] Valsecchi M., Aureli M., Mauri L., Illuzzi G., Chigorno V., Prinetti A., Sonnino S. (2010). Sphingolipidomics of A2780 human ovarian carcinoma cells treated with synthetic retinoids. J. Lipid Res..

[187] Sabichi A.L., Xu H., Fischer S., Zou C., Yang X., Steele V.E., Kelloff G.J., Lotan R., Clifford J.L. (2003). Retinoid receptor-dependent and independent biological activities of novel fenretinide analogues and metabolites. Clin. Cancer Res..

[188] Koh I., Jun H.-S., Choi J.S., Lim J.H., Kim W.H., Yoon J.B., Song J. (2012). Fenretinide ameliorates insulin resistance and fatty liver in obese mice. Biol. Pharm. Bull..

[189] Samuel W., Kutty R.K., Duncan T., Vijayasarathy C., Kuo B.C., Chapa K.M., Redmond T.M. (2014). Fenretinide induces ubiquitin-dependent proteasomal degradation of stearoyl-CoA desaturase in human retinal pigment epithelial cells. J. Cell. Physiol..

[190] Mody N., Mcilroy G.D. (2014). The mechanisms of Fenretinide-mediated anti-cancer activity and prevention of obesity and type-2 diabetes. Biochem. Pharmacol..

[191] Shearer K.D., Morrice N., Henderson C., Reekie J., Mcilroy G.D., McCaffery P.J., Delibegovic M., Mody N. (2015). Fenretinide prevents obesity in aged female mice in association with increased retinoid and estrogen signaling. Obesity.

[192] Morrice N., Mcilroy G.D., Tammireddy S.R., Reekie J., Shearer K.D., Doherty M.K., Delibegović M., Whitfield P.D., Mody N. (2017). Elevated Fibroblast growth factor 21 (FGF21) in obese, insulin resistant states is normalised by the synthetic retinoid Fenretinide in mice. Sci. Rep..

[193] Peng Y.M., Dalton W.S., Alberts D.S., Xu M.J., Lim H., Meyskens F.L. (1989). Pharmacokinetics of *N*-4-hydroxyphenyl-retinamide and the effect of its oral administration on plasma retinol concentrations in cancer patients. Int. J. Cancer.

[194] Costa A., Malone W., Perloff M., Buranelli F., Campa T., Dossena G., Magni A., Pizzichetta M., Andreoli C., Del Vecchio M. (1989). Tolerability of the synthetic retinoid Fenretinide (HPR). Eur. J. Cancer Clin. Oncol..

[195] Formelli F., Carsana R., Costa A., Buranelli F., Campa T., Dossena G., Magni A., Pizzichetta M. (1989). Plasma retinol level reduction by the synthetic retinoid fenretinide: A one year follow-up study of breast cancer patients. Cancer Res..

[196] Dimitrov N.V., Meyer C.J., Perloff M., Ruppenthal M.M., Phillipich M.J., Gilliland D., Malone W., Minn F.L. (1990). Alteration of retinol-binding-protein concentrations by the synthetic retinoid fenretinide in healthy human subjects. Am. J. Clin. Nutr..

[197] Decensi A., Torrisi R., Polizzi A., Gesi R., Brezzo V., Rolando M., Rondanina G., Orengo M.A., Formelli F., Costa A. (1994). Effect of the synthetic retinoid fenretinide on dark adaptation and the ocular surface. J. Natl. Cancer Inst..

[198] Decensi A., Fontana V., Fioretto M., Rondanina G., Torrisi R., Orengo M.A., Costa A. (1997). Long-term effects of fenretinide on retinal function. Eur. J. Cancer.

[199] Caruso R.C., Zujewski J., Iwata F., Podgor M.J., Conley B.A., Ayres L.M., Kaiser-Kupfer M.I. (1998). Effects of fenretinide (4-HPR) on dark adaptation. Arch. Ophthalmol..

[200] Conley B., O’Shaughnessy J., Prindiville S., Lawrence J., Chow C., Jones E., Merino M.J., Kaiser-Kupfer M.I., Caruso R.C., Podgor M. (2000). Pilot trial of the safety, tolerability, and retinoid levels of *N*-(4-hydroxyphenyl) retinamide in combination with tamoxifen in patients at high risk for developing invasive breast cancer. J. Clin. Oncol..

[201] Kurie J.M., Lee J.S., Khuri F.R., Mao L., Morice R.C., Lee J.J., Walsh G.L., Broxson A., Lippman S.M., Ro J.Y. (2000). *N*-(4-hydroxyphenyl)retinamide in the chemoprevention of squamous metaplasia and dysplasia of the bronchial epithelium. Clin. Cancer Res..

[202] Thaller C., Shalev M., Frolov A., Eichele G., Thompson T.C., Williams R.H., Dillioglugil O., Kadmon D. (2000). Fenretinide therapy in prostate cancer: Effects on tissue and serum retinoid concentration. J. Clin. Oncol..

[203] Baglietto L., Torrisi R., Arena G., Tosetti F., Gonzaga A.G., Pasquetti W., Robertson C., Decensi A. (2000). Ocular effects of fenretinide, a vitamin A analog, in a chemoprevention trial of bladder cancer. Cancer Detect. Prev..

[204] Abou-Issa H., Curley R.W., Alshafie G.A., Weiss K.L., Clagett-Dame M., Chapman J.S., Mershon S.M. (2001). Chemotherapeutic evaluation of 4-hydroxybenzylretinone (4-HBR), a nonhydrolyzable C-linked analog of *N*-(4-hydroxyphenyl) retinamide (4-HPR) against mammary carcinogenesis. Anticancer Res..

[205] Formelli F., Camerini T., Cavadini E., Appierto V., Villani M.G., Costa A., De Palo G., Di Mauro M.G., Veronesi U. (2003). Fenretinide breast cancer prevention trial: Drug and retinol plasma levels in relation to age and disease outcome. Cancer Epidemiol. Biomark. Prev..

[206] Sabichi A.L., Modiano M.R., Lee J.J., Peng Y.-M., Xu M.-J., Villar H., Dalton W.S., Lippman S.M. (2003). Breast tissue accumulation of retinamides in a randomized short-term study of fenretinide. Clin. Cancer Res..

[207] Garaventa A., Luksch R., Lo Piccolo M.S., Cavadini E., Montaldo P.G., Pizzitola M.R., Boni L., Ponzoni M., Decensi A., De Bernardi B. (2003). Phase I trial and pharmacokinetics of fenretinide in children with neuroblastoma. Clin. Cancer Res..

[208] Radu R.A., Han Y., Bui T.V., Nusinowitz S., Bok D., Lichter J., Widder K., Travis G.H., Mata N.L. (2005). Reductions in serum vitamin A arrest accumulation of toxic retinal fluorophores: A potential therapy for treatment of lipofuscin-based retinal diseases. Investig. Ophthalmol. Vis. Sci..

[209] Lippman S.M., Lee J.J., Martin J.W., El-Naggar A.K., Xu X., Shin D.M., Thomas M., Mao L., Fritsche H.A., Zhou X. (2006). Fenretinide activity in retinoid-resistant oral leukoplakia. Clin. Cancer Res..

[210] Colombo N., Formelli F., Cantù M.G., Parma G., Gasco M., Argusti A., Santinelli A., Montironi R., Cavadini E., Baglietto L. (2006). A phase I-II preoperative biomarker trial of fenretinide in ascitic ovarian cancer. Cancer Epidemiol. Biomark. Prev..

[211] Schneider B.J., Worden F.P., Gadgeel S.M., Parchment R.E., Hodges C.M., Zwiebel J., Dunn R.L., Wozniak A.J., Kraut M.J., Kalemkerian G.P. (2009). Phase II trial of fenretinide (NSC 374551) in patients with recurrent small cell lung cancer. Investig. New Drugs.

[212] Formelli F., Cavadini E., Luksch R., Garaventa A., Appierto V., Persiani S. (2010). Relationship among pharmacokinetics and pharmacodynamics of fenretinide and plasma retinol reduction in neuroblastoma patients. Cancer Chemother. Pharmacol..

[213] Chojkier M. A Randomized, Double-blind Study of the Effects of Fenretinide Administered in Subjects with Obesity. https://clinicaltrials.gov/ct2/show/NCT00546455.

[214] Oliveros L.B., Domeniconi M.A., Vega V.A., Gatica L.V., Brigada A.M., Gimenez M.S. (2007). Vitamin A deficiency modifies lipid metabolism in rat liver. Br. J. Nutr..

[215] Vega V.A., Anzulovich A.C., Varas S.M., Bonomi M.R., Giménez M.S., Oliveros L.B. (2009). Effect of nutritional vitamin A deficiency on lipid metabolism in the rat heart: Its relation to PPAR gene expression. Nutrition.

[216] Gatica L.V., Vega V.A., Zirulnik F., Oliveros L.B., Gimenez M.S. (2006). Alterations in the lipid metabolism of rat aorta: Effects of vitamin a deficiency. J. Vasc. Res..

[217] Kang H.W., Bhimidi G.R., Odom D.P., Brun P.-J., Fernandez M.-L., McGrane M.M. (2007). Altered lipid catabolism in the vitamin A deficient liver. Mol. Cell. Endocrinol..

